# ﻿New insights into *Lactocollybia* (Agaricales, Basidiomycota): Morpho-phylogenetic analyses revealing two interesting species and one new record from Thailand and evidence of intercontinental conspecificity

**DOI:** 10.3897/mycokeys.118.144986

**Published:** 2025-06-12

**Authors:** Ishika Bera, Komsit Wisitrassameewong, Naritsada Thongklang

**Affiliations:** 1 Center of Excellence in Fungal Research, Mae Fah Luang University, Chiang Rai 57100, Thailand; 2 Department of Biotechnology, Faculty of Science, Mahidol University, Bangkok 10400, Thailand; 3 School of Science, Mae Fah Luang University, Chiang Rai 57100, Thailand

**Keywords:** Agaricales, incertae sedis, phylogeny, species, taxonomy, Thailand

## Abstract

The genus *Lactocollybia* (*incertae sedis*, Agaricales) is a small and relatively understudied group of mushrooms, exhibiting unique morphological and ecological characteristics. Most species have a saprobic lifestyle, distributed in tropical to subtropical regions. Currently, the phylogenetic relationships of species remain poorly understood due to insufficient DNA sequence data of existing species. This study is an integrative approach, combining morphological characteristics and molecular analyses using nrITS and two-locus (nrITS-nrLSU) phylogenetic estimation. For the latter, we tested phylogenetic lineages of publicly available nrITS sequences and six samples from Thailand. Our phylogenies have revealed that Thai samples are placed in three phylogenetic clades, in which one of them is proposed as a new species, *L.polyhabitata*, whereas *L.variicystis* is reported for the first time from Thailand, suggesting a broader distribution of this species in the tropical region. Another species, L.cf.epia, has been critically analyzed for its affinity towards *L.epia*. All studied species are placed in phylogenetic clades with a multitude of collections from different continents, which suggests the evidence of intercontinental conspecificity across tropical and subtropical regions.

## ﻿Introduction

*Lactocollybia* Singer, currently classified as *incertae sedis*, Agaricales (Kalichman et al. 2020; Wijayawardene et al. 2022), is a widespread tropical to subtropical genus where species are recorded across both the southern and northern hemispheres. The genus is lignicolous or foliicolous, often found on plant debris, either solitary or gregarious, and is rarely terrestrial (Reid and Eicker 1998). The basidiomata can be collybioid, marasmioid, or mycenoid with an elastic texture (Pegler 1977; Reid and Eicker 1998) and are mostly white (exceptions: *L.aurantiaca* Singer, *L.dendrobii* Hauskn. & Krisai) (Singer and Digilio 1952; Hausknecht and Krisai-Greilhuber 2008). The members of this genus are characterized by a convex to plano-convex pileus, with or without a depressed center, a smooth to striate margin, more or less hygrophanous, adnexed to decurrent lamellae, a central and hollow stipe with a smooth to pruinose surface and without an annulus ring, occasional presence of latex, and a white spore deposit (Pegler 1977; Reid and Eicker 1998; Izhar et al. 2022). The micro-morphological characters are thin-walled, subglobose to amygdaliform inamyloid basidiospores; frequent presence of gloeocystidia and gloeohyphal elements in hymenium, pileipellis, and stipitipellis; and cutis nature of pileipellis (Pegler 1977).

The generic status and species delimitation in *Lactocollybia* are mainly based on morphological characters. The genus typified by *Collybialacrimosa* Heim [=*Lactocollybialacrimosa* (Heim) Singer] was first classified under Tricholomataceae R. Heim ex Pouzar by Singer (1939). Based on the resemblance with the genus *Macrocystidia* Joss in terms of the abundance of gloeocystidia and hymenial cystidia with inamyloid basidiospores, both genera were assumed to be closely related (Singer 1970, 1986). *Lactocollybia* was distinguished from *Macrocystidia* due to its white spore deposit and acyanophilic reaction of basidiospore walls (Singer 1986; Izhar et al. 2022). Later, *Lactocollybia* was transferred to Marasmiaceae Roze ex Kühner (Kirk et al. 2008) owing to the similar morphology and habitat and is currently considered as *incertae sedis* under the suborder Marasmiineae (Agaricales) (Kalichman et al. 2020). The genus has been divided into five sections based on the combination of morphological features such as habit and habitat, color of the basidiomata, nature of hymenophoral trama, types of gloeocystidia or gloeohyphal elements, and presence or absence of latex and clamp connections (Singer 1986, 1989). The recognized sections are *Bertrandiella* (Heim) Singer, *Lactocollybia* Singer, *Albae* Singer, *Aurantiacae* Singer, and *Graminicolae* Singer, as described by Singer (1986, 1989) and later supported by Izhar et al. (2022). Most of the species of this genus are regarded as pathogens frequently growing on the trunks of trees, while *L.aequatorialis* Singer is an exception for its culinary usage by Amazonian Indians (Singer 1986; Prance 1987; Putzke 2007). Currently, 17 species of *Lactocollybia* are accepted worldwide (Index Fungorum, MycoBank, accessed on 2.12.2024). A list of the species with their type locality is presented in Table 1.

**Table 1.** Type localities of currently accepted species of *Lactocollybia*.

Serial No.	Name of the species	Type locality	Reference
1.	*Lactocollybiaaequatorialis* Singer	Ecuador (South America)	Singer (1978)
2.	Lactocollybia*angiospermarum* Singer (considered as synonym of L.epia)	Florida (South America)	Singer (1948)
3.	*Lactocollybiaaurantiaca* Singer	Argentina (South America)	Singer and Digilio (1952)
4.	*Lactocollybiacarneipes* (Speg.) Singer	America	Singer (1986)
5.	*Lactocollybiadendrobii* Hauskn. & Krisai	Austria (Europe)	Hausknecht and Krisai-Greilhuber (2008)
6.	*Lactocollybiaepia* (Berk. & Broome)	Sri Lanka (South Asia)	Pegler (1986)
7.	*Lactocollybiaglobosa* D.A. Reid & Eicker	South Africa	Reid and Eicker (1998)
8.	*Lactocollybiagracillima* Pegler	(Tanzania) East Africa	Pegler (1977)
9.	*Lactocollybiagraminicola* Singer	Venezuela (South America)	Singer (1989)
10.	*Lactocollybiaholophaea* (Mont.) Singer	Jamaica (North America)	Singer (1973)
11.	*Lactocollybialacrimosa* (R. Heim) Singer	Europe	Singer (1939)
12.	*Lactocollybiamarasmiiformis* (Murrill) Singer	America	Singer (1986)
13.	*Lactocollybiamicrospora* Singer	Argentina (South America)	Singer (1962)
14.	*Lactocollybiamodesta* Singer	South America	Singer (1970)
15.	*Lactocollybiapiliicystis* D.A. Reid & Eicker	South Africa	Reid and Eicker (1998)
16.	*Lactocollybiasubvariicystis* Hosen & T.H. Li	China (East Asia)	Hosen et al. (2016)
17.	*Lactocollybiavariicystis* D.A. Reid & Eicker	South Africa	Reid and Eicker (1998)

Most *Lactocollybia* species are described based on morphology and ecology. Subtle morphology caused vague species delimitation and the proposal of illegitimate names. For example, the proposal to synonymize *L.angiospermarum* Singer and *L.cycadicola* (Joss.) Singer with *L.epia* (Berk. & Broome) was based on morphological similarity (Pegler 1977; Reid and Eicker 1998; Yang 2000). Many species have been documented with a wide distribution in tropical regions, such as *L.epia* (Sri Lanka, Kenya, Tanzania, China, and India), *L.globosa* D.A. Reid & Eicker, *L.gracillima* Pegler, and *L.piliicystis* D.A. Reid & Eicker (South Africa), *L.subvariicystis* (China), and *L.variicystis* (South Africa, São Tomé, India, Pakistan, and Iraq), which are yet to be authenticated (Hosen et al. 2016; Senthilarasu and Kumaresan 2016; Desjardin and Perry 2017; Yang 2000; Izhar et al. 2022; Al-Khesraji et al. 2022). DNA sequence data for *Lactocollybia* species are limited in public databases. Moreover, sequences of the holotype specimens are particularly scarce; to date, only for the holotype of *L.subvariicystis* has available DNA sequence. Many other sequences in public databases are either unidentified or misidentified, for example, those labeled as *L.aurantiaca* Singer, *L.epia*, and *L.variicystis* D.A. Reid & Eicker, making it difficult to assess their taxonomic placement in molecular phylogenetic analyses. Only two recent studies showed the phylogenetic placement of their studied species, and their analyses relied on the Internal Transcribed Spacer (nrITS) region solely (Hosen et al. 2016; Izhar et al. 2022). Thus, the knowledge of the evolutionary relationship of *Lactocollybia* species is poorly understood.

Thailand is part of the Indo-Burma biodiversity hotspot and harbors high biodiversity, making it one of the most species-rich areas in the world. The country has diverse ecosystems ranging from tropical rainforests and mangroves to mountainous regions and coastal areas. These ecosystems harbor a wide diversity of fungal species; however, they are largely underexplored, and many may represent interesting, yet undiscovered species (Hyde et al. 2018). It was reported that most of the previously studied fungal groups had a high percentage of species novelty and suggested that there are still many unknown species remaining to be described (Hyde et al. 2018). *Lactocollybia* has never been reported from Thailand. This study is the first documentation of this genus from the country. Macrofungal surveys during the monsoon season in various forested areas of Thailand revealed three interesting species of *Lactocollybia*. Thorough morphological examination and phylogenetic analyses have revealed one of them as new to science and one new addition to the Thai mycoflora. Lactocollybiacf.epia has been critically analyzed for its affinity towards *L.epia*. Due to the limited availability of nrITS and part of the 28S ribosomal RNA (nrLSU) sequences, morphological differences with the related and similar-looking species have been emphasized. Thus, the present study provides an overview of both the taxonomy and conspecific nature of the three described species from Thailand.

## ﻿Materials and methods

### ﻿Specimen acquisition

Six specimens were collected from two northern Thailand provinces, Lampang and Phrae, and one southern Thailand province, Narathiwat. The environment in northern Thailand has a tropical savanna climate supporting the semi-deciduous *Dipterocarpus* forests to evergreen forests. Both Lampang and Phrae experience tropical monsoon climates. Collections were conducted in dipterocarp forests dominated by *Dipterocarpusobtusifolius* Teijsm. ex Miq. and *D.tuberculatus* Roxb. in July and August. In Narathiwat province, specimens were collected from Princess Sirindhorn Wildlife Sanctuary (Pru To Daeng Wildlife Sanctuary) located near Malaysia’s southern border. This is the largest peat swamp forest in Thailand, a waterlogged tropical forest dominated by mangroves and trees belonging to Arecaceae. One of the specimens presented in this study was collected from this locality, inhabiting a submerged dead log in August.

### ﻿Morphological study

Macromorphological characters from immature to mature basidiomata were recorded in the forest and laboratory. Images of the fresh basidiomata were captured with a Nikon DSLR D3400 in the forest. Color codes and terms follow the Methuen Handbook of Color (Kornerup and Wanscher 1978). Chemical reaction tests with 5% KOH (potassium hydroxide) were done on the context of the specimens to observe color changes. After completion of the macro-characterization, the specimens were completely dried in an air dryer at 55 °C for 48 hours and then kept in paper bags. All micromorphological characteristics (basidia, hymenial cystidia, pileipellis elements, and basidiospores) were observed with an OLYMPUS BX-53 compound microscope. Freehand sections from dried specimens were mounted in distilled water (H_2_O), 5% KOH, or Melzer’s reagent to observe the gloeocystidial elements and determine the presence of pigments or color reactions. Sections from lamellae, pileus, and stipe were also mounted in 1% ammoniacal Congo Red after a short treatment with 5% KOH to observe the characters prominently. Micromorphological drawings were prepared with a drawing tube attached to the microscope at 1000× magnification. All measurements were taken with the help of CellSens Standard software dedicated to the OLYMPUS BX-53 microscope. The basidium length excludes sterigmata. Basidiospores were examined in Melzer’s reagent and 5% KOH and measured in side view. Thirty basidiospore measurements were recorded for each specimen. Basidiospore measurements and length/width ratios (Q) are recorded as minimum-*mean*-maximum. Thirty measurements for each of the other micromorphological characters were also recorded to have a range. Photomicrographs were taken with a camera attached to the compound microscope. After characterization, the holotype, paratype, and all the studied specimens are deposited in the Herbarium of Mae Fah Luang University (MFLU). MycoBank numbers were procured for the new species.

### ﻿DNA extraction, PCR amplification, and sequencing

The genomic DNA was isolated from dry herbarium specimens (approximately 50 mg per specimen) using the High Pure PCR Template Preparation Kit (Roche) following its protocol. The NanoDrop One Microvolume UV-Vis spectrophotometer (Thermo Scientific, USA) was utilized to assess both the quality and the quantity of DNA by measuring absorbance readings. The polymerase chain reaction (PCR) amplification of the nrITS and nrLSU was performed using the primer pairs ITS1-F and ITS4, and LR0R and LR5, respectively (White et al. 1990). PCR was carried out on a PCR thermal cycler (Eppendorf AG 22331 Hamburg Mastercycler) programmed for three minutes at 94 °C, followed by 35 cycles of 30 seconds at 95 °C, one minute at 55 °C, one minute at 72 °C, and a final extension step of ten minutes at 72 °C for the amplification of the nrITS and nrLSU regions. The sequencing of both strands of the PCR products was performed by SolGent Co., Ltd., Yuseong-gu, Daejeon, South Korea. The quality of the raw sequences was checked and edited manually where needed in BioEdit v.7.0.9 (Hall 1999) and assembled using SeqMan (DNAstar, Madison, WI, USA) and Geneious 9.1.2 (Kearse et al. 2012). The final consensus sequences were deposited at GenBank (https://www.ncbi.nlm.nih.gov/genbank/) to procure the accession numbers for nrITS (*L.variicystis*: PQ530286 and PQ530287, *L.polyhabitata*: PQ530288 and PQ530289, L.cf.epia: PQ530290 and PQ533676) and nrLSU (*L.variicystis*: PQ601632 and PQ601633, *L.polyhabitata*: PQ601634 and PQ601635, L.cf.epia: PQ601636 and PQ601637).

### ﻿Phylogenetic analyses

**Figure 1.**
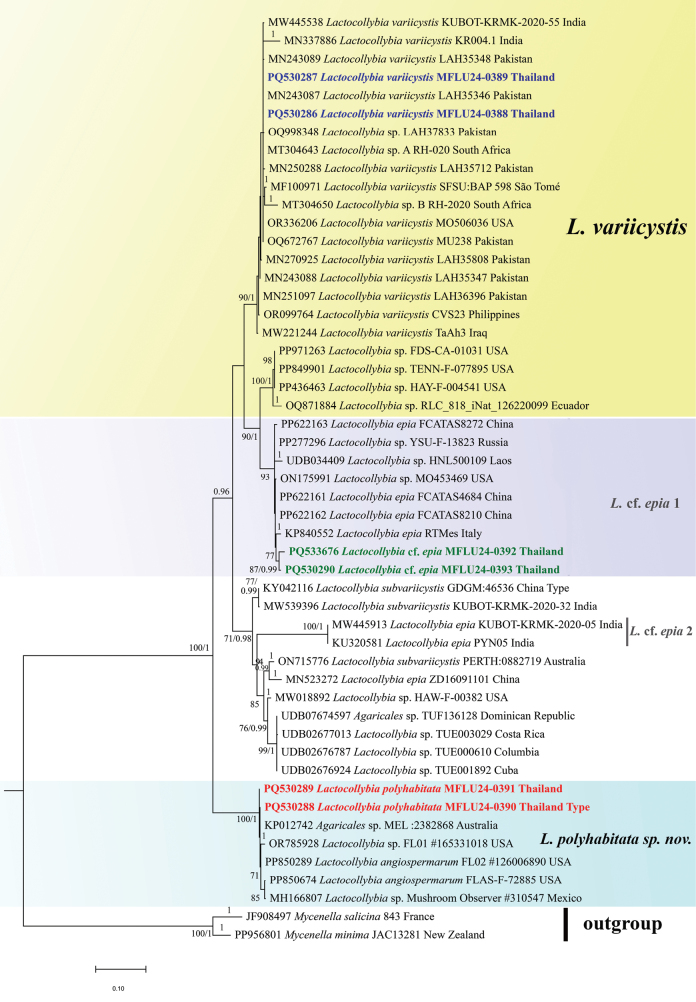
Phylogram inferred from maximum likelihood (ML) analysis by raxmlGUI 2.0 and Bayesian inference by Mr.Bayes v.3.2.6 based on nrITS sequence data. Generated sequences in this study are presented in red bold for the novel species and blue bold for the new record. Sequences of L.cf.epia are presented in green bold. The clade representing *L.variicystis*, *L.polyhabitata sp. nov.*, and L.cf.epia are demarcated by the yellow, blue, and purple boxes, respectively. Maximum likelihood bootstrap support values (MLB) ≥ 70% are shown on the left of “/,” and Bayesian posterior probabilities (BPP) ≥ 0.95 are shown on the right or below “/” at nodes.

To confirm the genus and identify the closest matches of the three species, Basic Local Alignment Search Tool (BLAST) analysis was performed for each sequence against the National Center for Biotechnology Information (NCBI) database (http://www.ncbi.nlm.nih.gov/genbank/). Table 2 shows the details of the sequences used in the phylogenetic analysis. To begin with, the phylogenetic analysis based on nrITS and nrLSU sequence data, two datasets (nrITS and nrLSU) were prepared separately, including the newly generated sequences, close relatives of the new taxa, available sequences of other species of the genus, and the outgroup species [*Mycenellaminima* Singer and *Mycenellasalicina* (Velen.) Singer] acquired from the BLAST search (Altschul et al. 1997) in GenBank (Clark et al. 2016), the UNITE database (Abarenkov et al. 2024), and relevant published phylogenies (Hosen et al. 2016; Izhar et al. 2022). These two datasets were then aligned using the online version of the multiple sequence alignment program MAFFT v7 (Katoh et al. 2019) with the iterative refinement methods as L-INS-i. The alignment was edited with trimAl 1.4.1 (Capella-Gutiérrez et al. 2009) to eliminate ambiguously aligned positions. The nrITS alignments were separated into three distinct partitions: ITS1, 5.8S, and ITS2. The selection of substitution models was accomplished using jModelTest 2.0 (Guindon and Gascuel 2003; Darriba et al. 2012). The best-fit models were HKY+G for ITS1, ITS2, and LSU and K80+G for 5.8S. Species delimitation was first examined using single-locus phylogenies. When no significant conflict was observed among single-locus phylogenies, we concatenated the two single-locus datasets (nrITS and nrLSU) into one dataset using Mesquite v.3.81 (Maddison and Maddison 2008). The nrITS and the concatenated nrITS-nrLSU sequences were separately analyzed using maximum likelihood (ML) and Bayesian inference (BI). Since only a few species have nrLSU sequences, the nrITS-based phylogenetic tree has also been presented for a better understanding of species delimitation (Fig. 1).

**Table 2.** Details of species used for the phylogenetic analysis in Figs 1, 2.

Species name	Voucher No.	Country	GenBank Numbers		References
			ITS	LSU	
Agariacles sp.	TUF136128	Dominican Republic	UDB07674597		UNITE direct submission
Agariacles sp.	MEL:2382868	Australia	KP012742		Direct Submission
*Lactocollybiaangiospermarum*	FL02, OMDL K. Canan iNaturalist # 126006890	USA: Florida	PP850289		Unpublished/Direct submission
*Lactocollybiaangiospermarum*	FLAS-F-72885	USA: Florida	PP850674		Unpublished/Direct submission
*Lactocollybiaepia*	RTMes	Italy	KP840552	KP840552	Unpublished/Direct submission
*Lactocollybiaepia*	ZD16091101	China	MN523272		Direct submission
*Lactocollybiaepia*	PYN05	India	KU320581		Direct submission
*Lactocollybiaepia*	FCATAS8272	China	PP622163		Direct Submission
*Lactocollybiaepia*	KUBOT-KRMK-2020-05	India	MW445913	MW442837	Unpublished/Direct submission
*Lactocollybiaepia*	FCATAS4684	China	PP622161		Direct Submission
*Lactocollybiaepia*	FCATAS8210	China	PP622162		Direct submission
***Lactocollybiapolyhabitata* Type**	**MFLU24-0390**	**Thailand**	**PQ530288**	**PQ601634**	**This study**
***Lactocollybiapolyhabitata***	**MFLU24-0391**	**Thailand**	**PQ530289**	**PQ601635**	**This study**
**Lactocollybiacfepia**	**MFLU24-0392**	**Thailand**	**PQ530290**	**PQ601636**	**This study**
**Lactocollybiacf.epia**	**MFLU24-0393**	**Thailand**	**PQ533676**	**PQ601637**	**This study**
*Lactocollybia* sp.	B RH-2020	South Africa	MT304650		Direct Submission
*Lactocollybia* sp.	A RH-2020	South Africa	MT304643		Unpublished
*Lactocollybia* sp.	FL01, OMDL K. Canan iNaturalist # 165331018	USA: Florida	OR785928		Unpublished/Direct submission
*Lactocollybia* sp.	Mushroom Observer # 310547	Mexico	MH166807		Direct Submission
*Lactocollybia* sp.	MO453469	USA: Ohio	ON175991		Unpublished/Direct submission
*Lactocollybia* sp.	YSU-F-13823	Russia	PP277296		Unpublished/Direct submission
*Lactocollybia* sp.	HAW-F-00382	USA	MW018892		Direct Submission (Mycoflora of Hawaii 2019)
*Lactocollybia* sp.	HAY-F-004541	USA: California	PP436463		Unpublished/Direct submission
*Lactocollybia* sp.	FDS-CA-01031	USA	PP971263		Direct Submission (CA FUNDIS)
*Lactocollybia* sp.	RLC_818_iNat_126220099	Ecuador	OQ871884		Vandegrift et al. (2023)
*Lactocollybia* sp.	TUE000610	Columbia	UDB02676787		UNITE direct submission
*Lactocollybia* sp.	TUE001892	Cuba	UDB02676924		UNITE direct submission
*Lactocollybia* sp.	TUE003029	Costa Rica	UDB02677013		UNITE direct submission
*Lactocollybia* sp.	TENN-F-077895	USA: California	PP849901		Unpublished/Direct submission
*Lactocollybia* sp.	HNL500109	Laos	UDB034409		UNITE direct submission
*Lactocollybia* sp.	LAH37833	Pakistan	OQ998348		Unpublished/Direct submission
*Lactocollybia* sp.	FG2018004	Australia		OL771804	Unpublished/Direct submission
*Lactocollybiasubvariicystis*	KUBOT-KRMK-2020-32	India	MW539396	MW538664	Unpublished/Direct submission
*Lactocollybiasubvariicystis*	PERTH:0882719	Australia	ON715776	ON715776	Unpublished/Direct submission
*Lactocollybiasubvariicystis*	GDGM:46535	China		KY042118	Hosen et al. (2016)
*Lactocollybiasubvariicystis* Type	GDGM:46536 Type	China	KY042116	KY042117	Hosen et al. (2016)
***Lactocollybiavariicystis***	**MFLU24-0388**	**Thailand**	**PQ530286**	**PQ601632**	**This study**
***Lactocollybiavariicystis***	**MFLU24-0389**	**Thailand**	**PQ530287**	**PQ601633**	**This study**
*Lactocollybiavariicystis*	MO506036	USA	OR336206		Direct Submission
*Lactocollybiavariicystis*	KR004.1	India	MN337886		Unpublished/Direct submission
*Lactocollybiavariicystis*	LAH35346	Pakistan	MN243087		Izhar et al. (2022)
*Lactocollybiavariicystis*	MU238	Pakistan	OQ672767		Direct Submission
*Lactocollybiavariicystis*	LAH35347	Pakistan	MN243088		Izhar et al. (2022)
*Lactocollybiavariicystis*	SFSU:BAP 598	São Tomé	MF100971		Desjardin and Perry (2017)
*Lactocollybiavariicystis*	CVS23	Philippines	OR099764		Unpublished/Direct submission
*Lactocollybiavariicystis*	KUBOT-KRMK-2020-55	India	MW445538		Unpublished/Direct submission
*Lactocollybiavariicystis*	LAH36396	Pakistan	MN251097		Izhar et al. (2022)
*Lactocollybiavariicystis*	LAH35348	Pakistan	MN243089		Izhar et al. (2022)
*Lactocollybiavariicystis*	LAH35712	Pakistan	MN250288		Izhar et al. (2022)
*Lactocollybiavariicystis*	LAH35808	Pakistan	MN270925		Izhar et al. (2022)
*Lactocollybiavariicystis*	TaAh3	Iraq	MW221244		Al-Khesraji et al. (2022)
*Mycenellaminima*	JAC13281	New Zealand	PP956801	PP956777	Unpublished/Direct submission
*Mycenellasalicina*	843	France	JF908497		Osmundson et al. (2013)

The ML analyses were performed in RAxMLGUI 2.0 (Edler et al. 2021) with the GTRGAMMA substitution model. ML analysis was executed by applying the thorough bootstrap algorithm with 1000 replicates to obtain nodal support values. Maximum likelihood bootstrap percentages (MLB) of 70% and above are considered significant support for clades.

The BI analyses were executed by employing four Markov chain Monte Carlo (MCMC) chains for a total of 1,000,000 generations, with termination criteria set at a standard deviation of split frequencies falling below the 0.01 threshold. Trees were sampled at every 100^th^ generation, with the initial 25% of trees being discarded as burn-in. The convergence of chains was assessed using Tracer 1.5 (Rambaut et al. 2018), ensuring that effective sample size (ESS) values exceeded 200, which confirmed the reliability of the results. In our phylogenetic analyses, gaps within the alignment were treated as missing data. Bayesian posterior probabilities (BPP) values of 0.95 and exceeding are considered strong support. The phylogenetic trees were observed in FigTree v. 1.4.4 (http://tree.bio.ed.ac.uk/software/figtree/) and were edited in Adobe Photoshop 2020 (Adobe Systems, USA).

## ﻿Results

### ﻿Phylogenetic inferences

**Figure 2.**
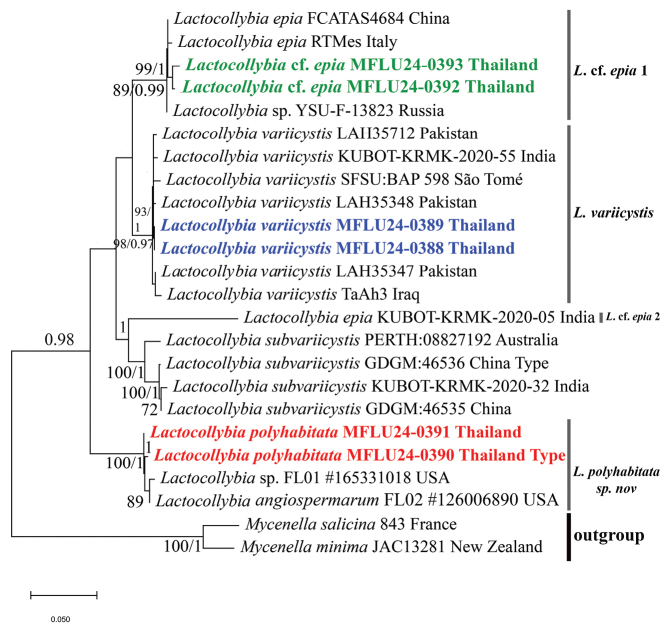
Phylogram inferred from maximum likelihood (ML) analysis by raxmlGUI 2.0 and Bayesian inference by Mr.Bayes v.3.2.6 based on nrITS-nrLSU sequence data. Generated sequences in this study are presented in red bold for the novel species and blue bold for the new record. Sequences of L.cf.epia are presented in green bold. Maximum likelihood bootstrap support values (MLB) ≥ 70% are shown on the left of “/,” and Bayesian posterior probabilities (BPP) ≥ 0.95 are shown on the right or below “/” at nodes.

The nrITS dataset (Fig. 1) for *Lactocollybia* includes 51 sequences representing nine species (with two outgroup species), six sequences of which are newly generated in this study. The nrITS alignment comprises 575 bases, including gaps. The final concatenated (nrITS-nrLSU) dataset (Fig. 2) consists of 24 sequences and 1460 characters, including gaps. The genus is monophyletic in both analyses (Figs 1, 2).

In the nrITS phylogenetic tree (Fig. 1), the two sequences of our studied *Lactocollybia* (PQ530286 and PQ530287) are well nested within the “*L.variicystis* clade,” representing sequences of *L.variicystis* from São Tomé, Pakistan, India, and Iraq, showing the conspecificity of Thai collections. It also showed similarity with other *L.variicystis* specimens from the Philippines, India, and the USA.

The sequences PQ530288 and PQ530289 are representing a separate clade and strongly clustered with two sequences named *L.angiospermarum* (PP850289, PP850674) and three sequences from unidentified sequences of *Lactocollybia*: one from the USA (OR785928), one from Australia (KP012742), and one from Mexico (MH166807). However, this whole clade is recovered as a separate species.

The specimens PQ530290 and PQ533676 are grouped in one of *L.epia* clades (designated as L.cf.epia 1). The Thai specimens are grouped with the sequences designated as *L.epia* (KP840552) from Italy, China (PP622161, PP622162, and PP622163), and some unidentified sequences from the USA (ON175991), Laos (UDB034409), and Russia (PP277296) (Fig. 1), while two Indian sequences are nested in L.cf.epia 2.

The nrITS-nrLSU concatenated tree (Fig. 2) exhibited the same relationship and topology as the nrITS tree, with the clades remaining consistent despite fewer sequences being included.

### ﻿Taxonomy

#### ﻿ Lactocollybia variicystis

Taxon classificationFungiAgaricalesMarasmiaceae

D.A. Reid & Eicker, Mycotaxon 66: 159

126B74B6-F3F9-547A-85A3-D091975E3555

Figs 3 , 4

##### Description.

***Basidiomata*** small-sized, collybioid. ***Pileus*** 5–15 mm diam., convex when young, gradually becoming planoconvex on maturity; surface moist, smooth, hygrophanous; white to yellowish white (1A1–2), changing to greyish yellow (1–2B4) on drying or bruising; margin entire or rarely undulating, decurved. ***Lamellae*** adnate, white (1A1) changing to greyish yellow (1B4) on drying or bruising, crowded (25 L+l/cm at pileus margin); lamellulae present in 6 series; edge entire. ***Stipe*** 7.5–22.5 × 1.5–2.5 mm, mostly central to slightly eccentric, cylindrical; surface moist, smooth, hygrophanous; white to yellowish white (1A1–2), changing to greyish yellow (1–2B4–5) and darker on drying or bruising; basal mycelium white. ***Context*** in pileus up to 1.3 mm thick, white (1A1), unchanging on bruising, exposure, and in 3% KOH; hollow in stipe, yellowish white (1A2), unchanging on bruising, exposure, and in 3% KOH.

**Figure 3.**
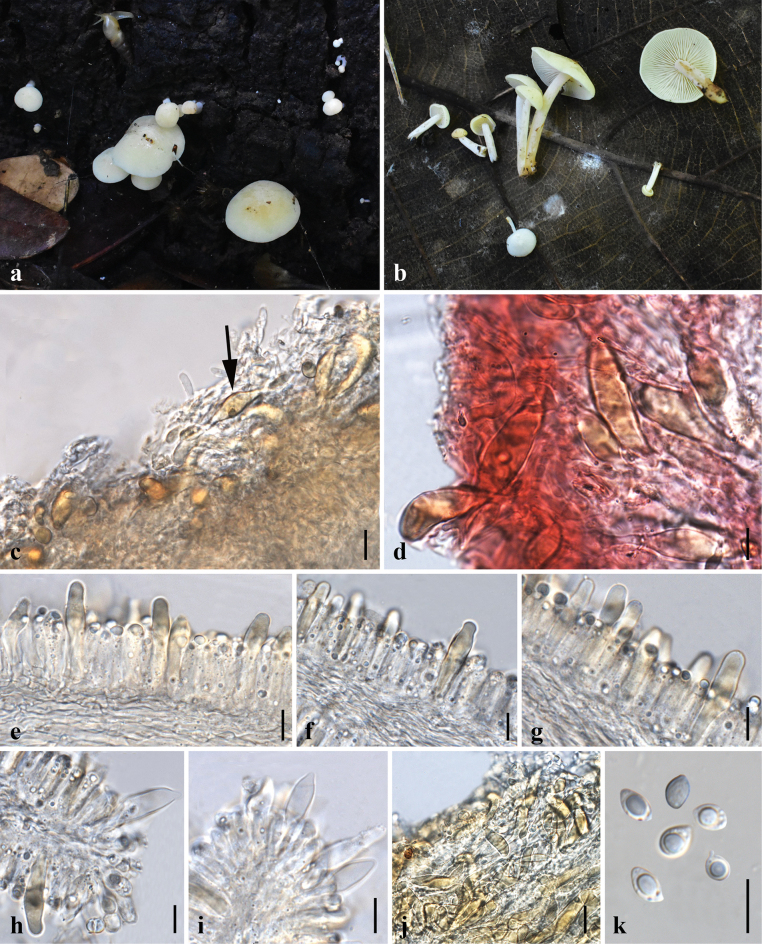
Morphological features of *Lactocollybiavariicystis* (MFLU24-0388) **a, b** fresh basidiomata in the field **c** pileipellis in 5% KOH **d** transverse section of pileipellis **e–g** gloeocystidia in hymenium in 5% KOH **h, i** cheilocystidia **j** transverse section through stipitipellis **k** basidiospore. Scale bars: 10 μm (**d–k**); 25 μm (**c**).

**Figure 4.**
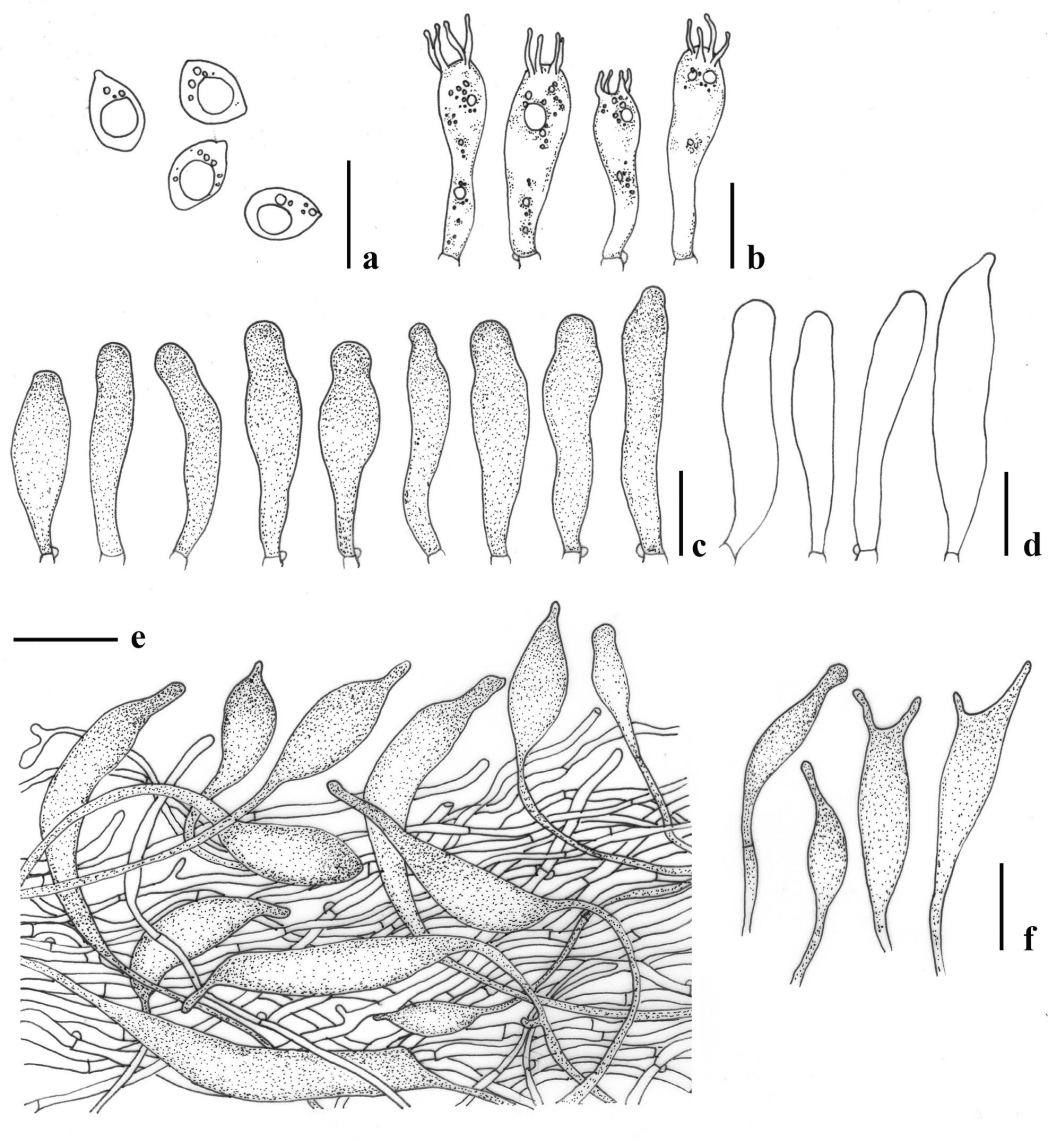
Micromorphological features of *Lactocollybiavariicystis* (MFLU24-0388) **a** basidiospores **b** basidia **c** gloeocystidia in hymenium **d** cheilocystidia **e** transverse section through pileipellis **f** gloeocystidia in stipe. Scale bars: 10 μm (**a–f**).

***Basidiospores*** (3.1)4.4–5.4–6.9 × (2.3)3.1–3.9–4.5(5.1) μm [n = 30, Q = (1.04)1.18–1.38–1.62(1.7), ellipsoid, rarely subglobose; thin-walled, smooth, apiculate, uni-guttulate, hyaline in 5% KOH, inamyloid, non-dextrinoid. ***Basidia*** 20.7–23 × 4.8–6.2 μm, clavate to subclavate, thin-walled, hyaline in 5% KOH, non-dextrinoid, 4-spored; sterigmata up to 5.5 μm long. ***Gloeocystidia*** abundant, more abundant on lamellae side, 24–32.6 × 3.2–6.2 μm, cylindrical to subcylindrical with mostly obtuse to sub-capitate apices, thin-walled, arising from the hymenophoral trama, yellowish brown in H_2_O and 5% KOH, non-dextrinoid; emergent up to 6 μm. ***Pleurocystidia*** absent. ***Lamellae edge*** fertile, heteromorphous with basidia, basidioles, and cystidia. ***Cheilocystidia*** abundant, 22.4–34.3 × 4.4–6.8 μm, cylindrical to subcylindrical with mostly obtuse to sometimes sub-fusoid, sub-capitate to mucronate apices, thin-walled, hyaline in 5% KOH, non-dextrinoid; emergent up to 13 μm. ***Subhymenium*** thin, up to 13 μm thick, subcellular with ramifying hyphae. ***Hymenophoral trama*** composed of compactly arranged, subparallel to parallel, thin-walled, septate hyphae and gloeohyphal elements; hyphae 3.5–6 μm wide. ***Pileipellis*** a cutis with interspersed pale brownish pigment; composed of shortly catenulate, sometimes branched hyphae with numerous scattered gloeocystidia; hyphae 2.4–4.5 μm wide with obtuse, sub-capitate to sub-fusoid apices, thin-walled, septate, hyaline in 5% KOH, non-dextrinoid; gloeocystidia lageniform to fusoid, 26.5–65 × 7.6–10 μm, mostly attenuated at both ends, refractive, yellowish in H_2_O and 5% KOH. ***Pileus trama*** composed of compactly arranged, interwoven hyphae and gloeohyphal elements; hyphae hyaline in 5% KOH, non-dextrinoid. ***Stipitipellis*** a cutis; composed of uprising hyphae with numerous scattered gloeocystidia; hyphae 2.2–4 μm wide with obtuse to sub-capitate apices, thin-walled, septate, hyaline in 5% KOH, non-dextrinoid; gloeocystidia lageniform to fusoid, 17.8–31.2 × 3.5–6.1 μm, often forked at apices, attenuated at base, refractive, yellowish in H_2_O and 5% KOH. ***Stipe trama*** composed of compactly arranged, parallel hyphae and gloeohyphal elements; hyphae hyaline in 5% KOH, non-dextrinoid. ***Clamp connections*** common.

##### Materials examined.

Thailand • Lampang Province: Mueang Lampang district, 18°21.79314'N, 99°17.05644'E, Alt. 399 m, gregarious on a *Dipterocarpus* sp. tree in semi-deciduous *Dipterocarpus* dominated forest, 18^th^ June 2023, *I. Bera*, IB 23-L02 (MFLU24-0388); 18°21.80328'N, 99°17.0535'E, Alt. 402 m, gregarious on a *Dipterocarpus* sp. tree in semi-deciduous *Dipterocarpus* dominated forest, 23^rd^ August 2023, *I. Bera*, IB 23-L06 (MFLU24-0389).

##### Notes.

The Thai *Lactocollybiavariicystis* is characterized by its small-statured, smooth, and hygrophanous, white to yellowish-white pileus; adnate and crowded lamellae; uni-guttulate ellipsoid basidiospores; omnipresence of yellowish-brown gloeocystidia in the hymenium; pale brownish pigmented pileipellis; and numerous scattered lageniform to fusoid and yellowish gloeocystidia in the pileipellis and stipitipellis. Additionally, the greyish yellow color change on bruising or drying makes this species quite distinct. The subcellular with ramifying hyphal nature of subhymenium and abundant gloeocystidia and clamp connection undoubtedly placed this species in the sect. Albae (Singer 1986).

In the phylogenetic analysis (Fig. 1), inclusion of two sequences retrieved from our collections (PQ530286 and PQ530287) within this clade suggests the identification of Thai sequences as *L.variicystis*. In that case, PQ530286 and PQ530287 represent the new record of *L.variicystis* from Thailand.

The type specimen of *L.variicystis* was discovered from *Salix* stump in South Africa (Reid and Eicker 1998). Our specimens are similar to the type specimen in respect of the size and color of the basidiomata, hymenial gloeocystidia, and cheilocystidia (Reid and Eicker 1998). However, we found differences in some characters compared to the type specimen. The striate or plicate margin, adnexed attachment of lamellae, furcation in lamellae at margin sometimes, slightly larger (6.6–8 × 4–6 μm) and broadly amygdaliform basidiospores, quite larger gloeocystidia (150 × 11.6 μm), wider hyphae (4–11.6 μm) in pileipellis, and presence of caulocystidia of the type specimen (Reid and Eicker 1998) differentiated it from our studied *Lactocollybia* species. Certain morphological details, such as changes in basidiomata color upon bruising, lamellae spacing, and corresponding molecular data of the type specimen, are lacking for comparison with our studied *L.variicystis*.

#### ﻿ Lactocollybia polyhabitata

Taxon classificationFungiAgaricalesMarasmiaceae

I. Bera sp. nov.

7CA5E51F-23B2-527B-A75A-6CF35B78A8DB

856767

Figs 5 , 6

##### Diagnosis.

The ellipsoid to oblong, uni- to multi-guttulate basidiospores and absence of hymenial gloeocystidia distinguish this *Lactocollybia* species.

##### Type.

Thailand • Narathiwat Province: Princess Sirindhorn Wildlife Sanctuary, N 6°4.4388'N, 101°58.14594'E, Alt. 30 m, gregarious on a dead log submerged in the water in a peat swamp forest, 4^th^ August 2023, *I. Bera*, IB 23-N15 (MFLU24-0390, holotype!)

##### Etymology.

The epithet ‘*polyhabitata*’ refers to the occurrence of the species across diverse habitat types, ranging from peat swamp forests to tropical forests.

##### Description.

***Basidiomata*** small-sized, collybioid. ***Pileus*** 6–16 mm diam., planoconvex when young, gradually becoming applanate on maturity; surface dry, minutely pruinose, hygrophanous; yellowish white (1A2), sometimes with greyish yellow (4B3) patches near the center; margin entire to undulate, decurved. ***Lamellae*** adnate, yellowish white (1A2), crowded (37 L+l/cm at pileus margin); lamellulae present in 4–5 series; edge entire to eroded. ***Stipe*** 10.4–15.7 × 1.3–2.2 mm, eccentric, cylindrical but tapering towards base; surface dry, smooth, hygrophanous; yellowish white (1A2) at apex gradually becoming pale yellow to light yellow (4A3–5) at base; basal mycelium white. ***Context*** in pileus up to 1.8 mm thick, white (1A1), unchanged on bruising, exposure, and in 3% KOH; hollow in stipe, yellowish white (1A2), unchanging on bruising, exposure, and in 3% KOH.

**Figure 5.**
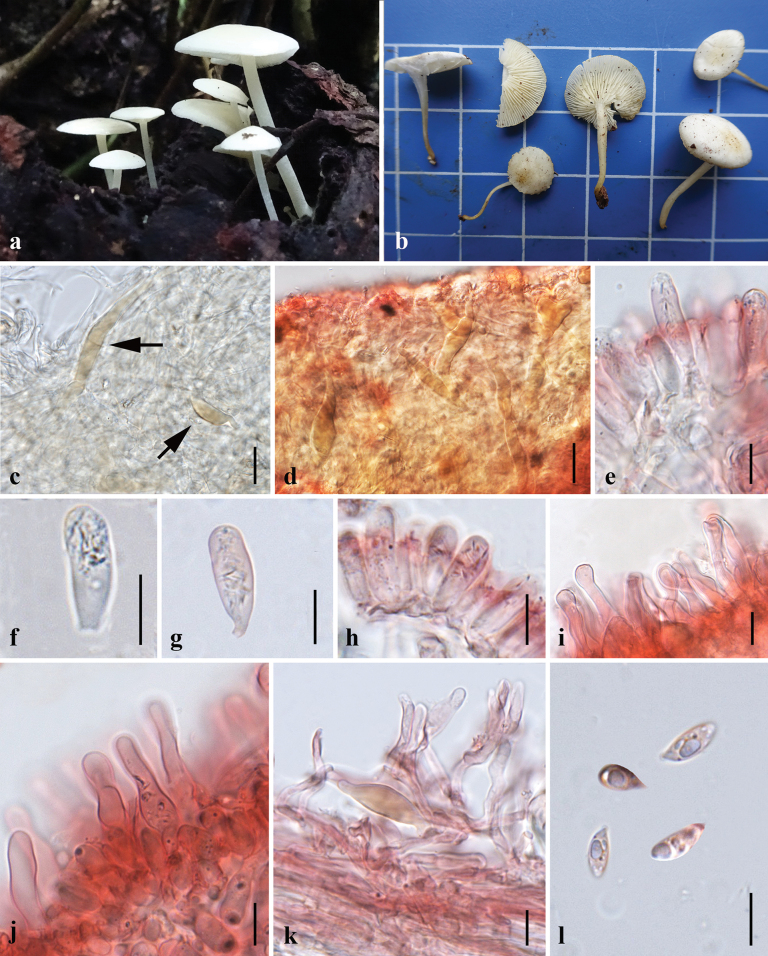
Morphological features of *Lactocollybiapolyhabitata* (MFLU24-0390, holotype) **a, b** fresh basidiomata in the field **c** pileipellis in 5% KOH **d** transverse section of pileipellis **e–h** basidioles with crystalline content **i, j** cheilocystidia **k** transverse section through stipitipellis **l** basidiospore. Scale bars: 20 μm (**c, d, i**); 10 μm (**e–h, j–l**).

***Basidiospores*** 6.3–8.2–10.6 × 3.4–4.1–4.9 μm [n = 30, Q = 1.48–2.01–2.62], ellipsoid to oblong; thin-walled, smooth, apiculate, uni- to multi-guttulate, hyaline in 5% KOH, inamyloid, non-dextrinoid. ***Basidia*** 22.1–29.2 × 4.6–6.6 μm, subclavate, thin-walled, hyaline in 5% KOH, non-dextrinoid, 4-spored; sterigmata up to 3.8 μm long. ***Basidioles*** 14.5–27.5 × 4.6–5.8 μm, subclavate, thin-walled, hyaline in 5% KOH, non-dextrinoid; sometimes have crystalline content. ***Lamellae edge*** fertile, heteromorphous with basidia, basidioles, and cystidia. ***Pleurocystidia*** absent. ***Hymenial gloeocystidia*** absent. ***Cheilocystidia*** abundant, 15.6–36.6 × 2.5–6.2 μm, variable in shape from subcylindrical, subclavate to lageniform with obtuse to sub-capitate apices, sometimes with swollen bases abruptly tapering towards apices forming undulating long necks, thin-walled, hyaline in 5% KOH; content rare, crystalline; emergent up to 20 μm. ***Subhymenium*** thin, up to 10 μm thick, subcellular with ramifying hyphae. ***Hymenophoral trama*** composed of compactly arranged, subparallel to parallel, thin-walled, septate hyphae; hyphae up to 3.5 μm wide. ***Pileipellis*** a cutis; composed of loosely interwoven, septate hyphae with numerous scattered long, fusoid gloeohyphal elements; hyphae 1.7–3.2 μm wide, thin-walled, septate, hyaline in 5% KOH, non-dextrinoid; gloeohyphal elements 22–95 × 8–19.2 μm, attenuated at both ends, refractive, yellowish in H_2_O and 5% KOH. ***Pileus trama*** composed of compactly arranged, interwoven hyphae and gloeohyphal elements; hyphae hyaline in 5% KOH, non-dextrinoid. ***Stipitipellis*** a cutis; composed of loosely interwoven, uprising hyphae with numerous scattered gloeocystidia and caulocystidia; hyphae 1.5–2.3 μm wide, thin-walled, septate, hyaline in 5% KOH, non-dextrinoid; gloeocystidia lageniform, 13.5–45.7 × 4–9.2 μm, attenuated at both ends, refractive, yellowish in H_2_O and 5% KOH; caulocystidia 19.2–21.6 × 3.2–6 μm, similar to cheilocystidia but shorter. ***Stipe trama*** similar to pileus trama, composed of compactly arranged, parallel hyphae and gloeohyphal elements; hyphae hyaline in 5% KOH, non-dextrinoid. ***Clamp connections*** common.

**Figure 6.**
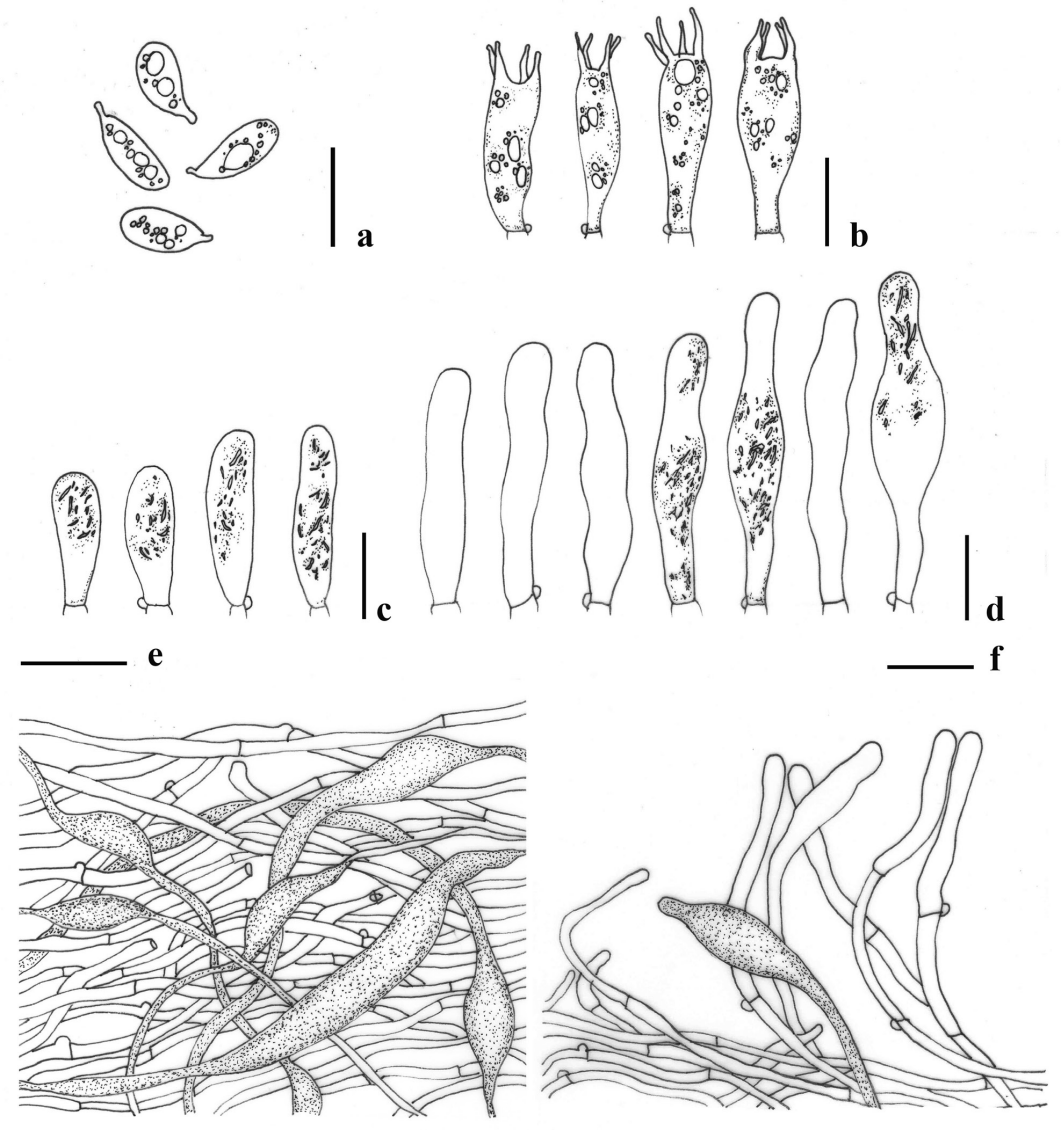
Micromorphological features of *Lactocollybiapolyhabitata* (MFLU24-0390, holotype) **a** basidiospore **b** basidia **c** basidiole with crystalline content **d** cheilocystidia **e** transverse section through pileipellis **f** transverse section through stipitipellis showing gloeocystidia and caulocystidia in stipe. Scale bars: 10 μm (**a–f**).

##### Additional material examined.

Thailand • Phrae Province: roadside, 18°10.75188'N, 100°10.82418'E, Alt. 171 m, gregarious on a dead tree log in semi-deciduous *Dipterocarpus* dominated forest, 3^rd^ August 2024, *I. Bera*, IB 24-47 (MFLU24-0391, paratype).

##### Notes.

*Lactocollybiapolyhabitata* belongs to the sect. Albae due to its subcellular structure with ramifying hyphae in the subhymenium, presence of gloeohyphal elements, and clamp connections (Singer 1986). The species can be confused with other species by typical field characters such as small-sized and yellowish-white basidiomata, hygrophanous and pruinose surfaces, and crowded lamellae. However, it can be distinguished by microscopic characters such as the presence of small cheilocystidia (with crystalline content) and caulocystidia and the absence of pleurocystidia and hymenial gloeocystidia. The species is found in various habitats at low elevations (30–171 m above sea level), including peat swamp forests and *Dipterocarpus* dominated forests.

Nearly all *Lactocollybia* species possess prominent hymenial gloeocystidia, readily distinguishing *L.polyhabitata* (Singer and Digilio 1952; Pegler 1977, 1986; Singer 1989; Reid and Eicker 1998). However, this character makes it similar to a few species, the African *L.gracillima* (Pegler 1977) and Chinese *L.subvariicystis* (Hosen et al. 2016). *Lactocollybiagracillima* differs by its transparent striations almost reaching the pileus center, decurrent lamellae, smaller basidiospores (5.3–7.3 × 2.7–3.7 μm), clavate-cylindric cheilocystidia with subcapitate to rounded apices, and caulocystidia with refractive contents (Pegler 1977). *Lactocollybiasubvariicystis* is differentiated by adnexed to sinuate lamellae attachment, amygdaliform to fusoid, pale yellowish basidiospores, the presence of pleurocystidia, and fusoid to subfusoid or lageniform with long-necked cheilocystidia, easily separating from *L.polyhabitata*.

The oblong basidiospore of *L.polyhabitata* also makes it unique. This character easily distinguishes it from other species with white basidiomata, such as *L.subvariicystis* (amygdaliform to broadly fusoid), *L.globosa* (ovoid to subglobose to tear-shaped), *L.piliicystis* (amygdaliform), *L.variicystis* (broadly amygdaliform), *L.microspora* (ellipsoid), and *L.gracillima* (ellipsoid to lacrymoid) (Singer 1962; Pegler 1977; Reid and Eicker 1998; Hosen et al. 2016). Though a similarly shaped basidiospore is reported in *L.epia* (as elongate-ellipsoid or fusoid), the presence of hymenial gloeocystidia and fine granular surface incrustations of pileus hyphae separates this species from *L.polyhabitata* (Pegler 1977, 1986).

Phylogenetically, nrITS sequences of our samples (PQ530288–PQ530289) clustered with three sequences designated as *L.angiospermarum* and three unidentified sequences (KP012742, OR785928, and MH166807) with strong support (MLB 100 and BPP 1, Fig. 1). *Lactocollybiaangiospermarum* was originally found in the USA by Singer (1948) and subsequently reported in East Africa by Pegler (1977). The species has been considered as a synonym of *L.epia* by various authors (Pegler 1986; Reid and Eicker 1998; Yang 2000). According to the protologue of *L.angiospermarum* and *L.epia* and the description of *L.angiospermarum* written by Pegler (1977), the morphology of both species is similar (Table 4). We could not assess the conspecificity of both species molecularly in this study. The public sequences designated for both species in this study lack morphological data. The sequences of *L.angiospermarum* (MH166807, PP850289, and PP850674) did not cluster with public *L.epia* sequences [labeled as L.cf.epia 1 and L.cf.epia 2 clades in this study (Fig. 1)]. At this stage, based on the available morphological data of both species, we agree that *L.angiospermarum* could be considered as the synonym of *L.epia*. The additional samples from the type locality coupled with morphological data would be helpful in taxonomic reassessment of both species.

*Lactocollybiapolyhabitata* differs from both *L.epia* and *L.angiospermarum* by having ellipsoid to oblong basidiospores (Q = 1.48–2.62), the absence of gloeocystidia in the hymenium, and yellowish gloeohyphal content (Table 4). Thus, this species is quite different based on the morphological distinction.

#### ﻿ Lactocollybia cf. epia

Taxon classificationFungiAgaricalesMarasmiaceae

6DD9C2B3-955B-5E33-9DD9-937A0CF6D993

Figs 7 , 8

##### Description.

***Basidiomata*** small-sized, collybioid. ***Pileus*** 4–28 mm diam., broadly convex to planoconvex when young, gradually becoming applanate on maturity; surface moist, smooth, translucent-striate up to 2/3^rd^ of the pileus from margin, hygrophanous; white to yellowish white (1A1–2) and translucent when wet, changing to greyish yellow (2B3) to the dried and bruised areas; margin entire to undulating, decurved. ***Lamellae*** adnate to subdecurrent, white (1A1) changing to greyish yellow (2B4) on drying and bruising, crowded (31 L+l/cm at pileus margin); lamellulae present in 5 series; edge entire to eroded. ***Stipe*** 10–15 × 1.5–2.5 mm, slightly eccentric, cylindrical; surface moist, smooth, hygrophanous; white (1A1) and translucent when moist but changing to greyish yellow (2B4) on drying and bruising; basal mycelium white. ***Context*** in pileus up to 1.5 mm thick, white (1A1), unchanged on bruising, exposure, and in 3% KOH; hollow in stipe, yellowish white (1A2), unchanging on bruising, exposure, and in 3% KOH.

**Figure 7.**
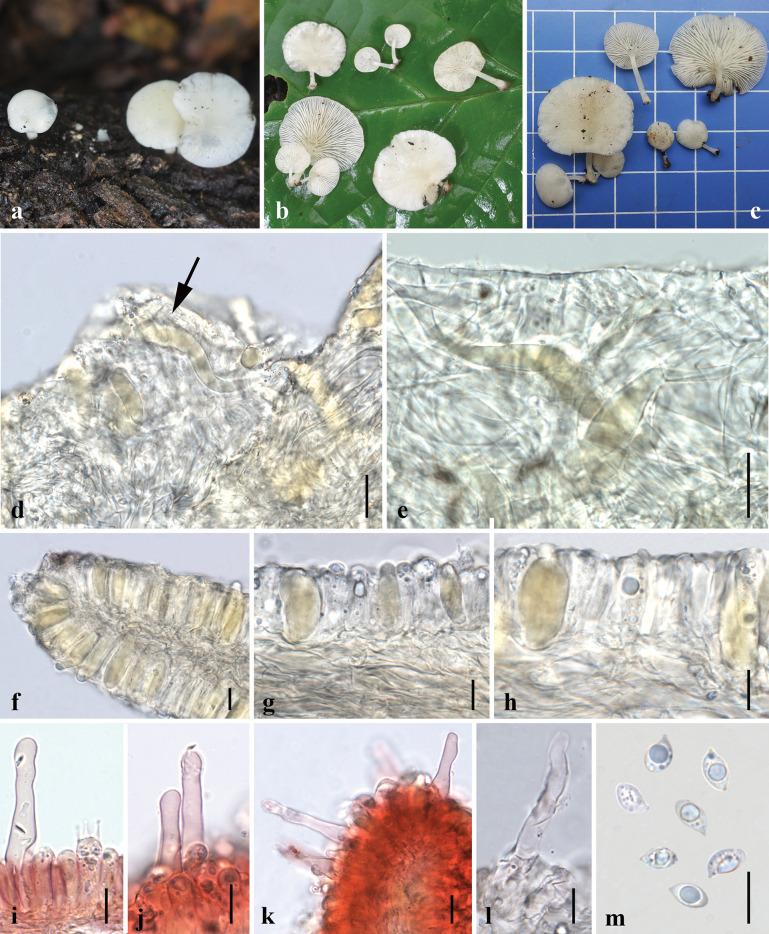
Morphological features of Lactocollybiacf.epia (MFLU24-0392) **a–c** fresh basidiomata in the field **d, e** transverse section of pileipellis **f–h** gloeocystidia in hymenium **i**, **j** pleurocystidia **k** cheilocystidia **l** caulocystidia **m** basidiospore. Scale bars: 20 μm (**d, e**); 10 μm (**f–m**).

***Basidiospores*** 5.3–6.9–8.4 × 3.9–4.4–4.7 μm [n = 40, Q = 1.25–1.55–1.84], broadly ellipsoid to subamygdaliform; thin-walled, smooth, apiculate, uni- to multi-guttulate, hyaline in 5% KOH, inamyloid, non-dextrinoid. ***Basidia*** 19.8–23.9 × 5.6–7.8 μm, subclavate, thin-walled, hyaline in 5% KOH, non-dextrinoid, 2- or 4-spored; sterigmata up to 4.6 μm long. ***Gloeocystidia*** abundant in both lamellae edge and lamellae side, 17.8–28.9 × 6–12.9 μm, mostly ovoid to subcylindrical, inflated, or clavate, sometimes subventricose or subfusoid, thin-walled, yellowish in H_2_O and 5% KOH, non-dextrinoid; originating from hymenium and subhymenium; non-emergent. ***Pleurocystidia*** moderate, 45.1–58.5 × 6.9–9.1 μm, cylindrical with generally obtuse apices and rarely sub-mucronate apices, slightly undulating near apex and sometimes with long neck, thin-walled, hyaline in 5% KOH, non-dextrinoid; content rare, crystalline; emergent up to 32 μm. ***Lamellae edge*** fertile, heteromorphous with basidia, basidioles, and cystidia. ***Cheilocystidia*** moderate, 23.4–53.5 × 4.1–7.6 μm, similar to pleurocystidia; emergent up to 33.6 μm. ***Subhymenium*** thin, up to 11.8 μm thick, subcellular with ramifying hyphae. ***Hymenophoral trama*** composed of compactly arranged, subparallel to parallel, thin-walled, septate hyphae and gloeohyphal elements; hyphae up to 4.8 μm wide. ***Pileipellis*** a cutis; composed of interwoven, septate hyphae with numerous scattered long, fusoid gloeohyphal elements; hyphae 4.2–6.9 μm wide, thin-walled, septate, hyaline in 5% KOH, non-dextrinoid; gloeohyphal elements 23.2–67 × 6–11.5 μm, attenuated at both ends, refractive, yellowish in H_2_O and 5% KOH, non-dextrinoid. ***Pileus trama*** composed of compactly arranged, loosely interwoven hyphae and gloeohyphal elements; hyphae hyaline in 5% KOH, non-dextrinoid. ***Stipitipellis*** a cutis; composed of parallelly arranged hyphae with scattered caulocystidia; hyphae 1.7–3.5 μm wide, thin-walled, septate, hyaline in 5% KOH, non-dextrinoid; caulocystidia 19.8–70.2 × 3–6.4 μm, similar to pleurocystidia and cheilocystidia. ***Stipe trama*** similar to pileus trama, composed of compactly arranged, parallel hyphae and gloeohyphal elements; hyphae hyaline in 5% KOH, non-dextrinoid. ***Clamp connections*** common.

**Figure 8.**
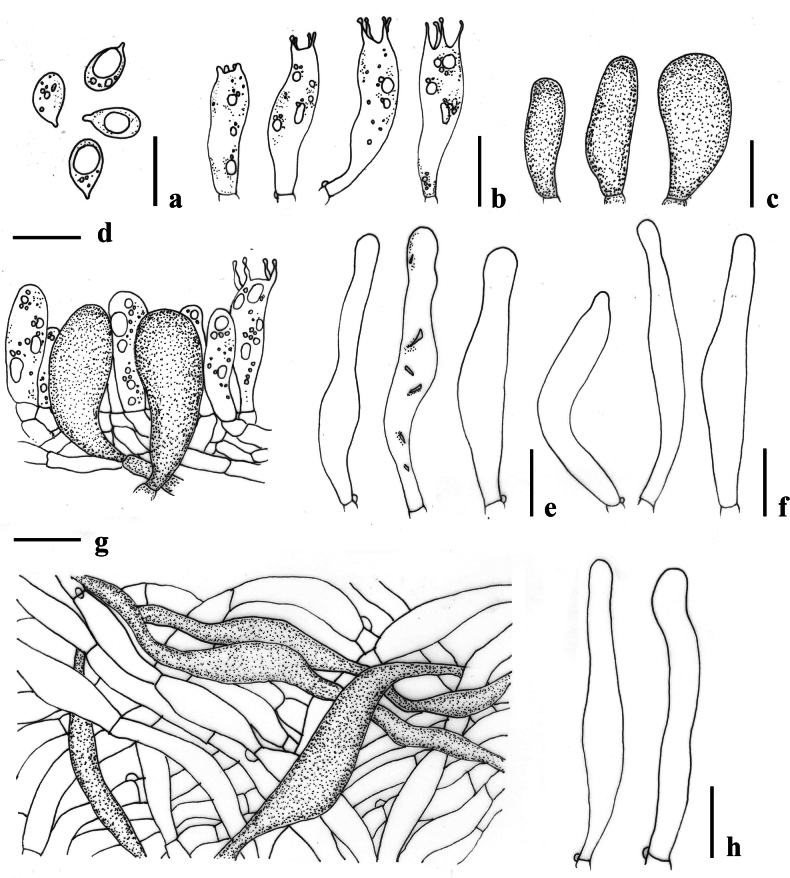
Micromorphological features of Lactocollybiacf.epia (MFLU24-0392) **a** basidiospores **b** basidia **c, d** gloeocystidia in hymenium **e** pleurocystidia **f** cheilocystidia **g** transverse section through pileipellis **h** caulocystidia. Scale bars: 10 μm (**a–h**).

##### Materials examined.

Thailand • Phrae Province: Rong Kwang roadside, 18°22.28712'N, 100°22.4661'E, Alt. 309 m, gregarious on a dead tree log in a semi-deciduous *Dipterocarpus*-dominated forest, 2^nd^ August 2024, *S. Khyaju*, IB 24-38 (MFLU24-0392); 18°10.1661'N, 100°2.43906'E, Alt. 312 m, gregarious on a dead tree log in a semi-deciduous *Dipterocarpus*-dominated forest, 3^rd^ August 2024, *I. Bera*, IB 24-67 (MFLU24-0393)

##### Notes.

This species of Lactocollybia belongs to the sect. Albae (Singer 1986). Lactocollybiacf.epia is characterized by its white to yellowish-white, hygrophanous basidiomata turning greyish-yellow on drying or bruising; translucent striations around the pileus margin; adnate to subdecurrent and crowded lamellae; broadly ellipsoid to subamygdaliform, uni- to multi-guttulate basidiospores; abundant gloeocystidia in both the lamellae edge and lamellae side; the presence of hymenial and pileal cystidia; abundant gloeohyphal elements in the pileus and stipe trama; and clamp connections.

In the phylogenetic inference (ML and BI) depicted in Fig. 1, the sequences generated from the collections of this Thai *Lactocollybia* reveal their relatedness to sequences labelled as *L.epia* from Italy and China, along with some unidentified *Lactocollybia* sequences from the USA, Laos, and Russia. All these sequences are from undescribed specimens submitted to the database. *Lactocollybiaepia* was originally described from Sri Lanka (Pegler 1986). Afterwards, *L.epia* has been reported from various countries, e.g., South Africa (Reid and Eicker 1998), China (Yang 2000), Brazil (Cortez and Sulzbacher 2009), and India (Senthilarasu and Kumaresan 2016). Unfortunately, the identification of the holotype and other samples in the published articles lacks molecular verification. Based on the morphology in the protologue and recent descriptions of *L.epia*, the species possesses differently shaped hymenial gloeocystidia (clavate fusiform with mucronate apices) with greenish yellow content, larger gloeohyphal elements, a complete absence of pleurocystidia, and fine granular surface incrustations of pileus hyphae (Pegler 1977, 1986; Reid and Eicker 1998; Yang 2000; Cortez and Sulzbacher 2009; Senthilarasu and Kumaresan 2016). However, shorter and yellowish content of gloeohyphal elements and prominent presence of pleurocystidia make our studied species different from *L.epia* (Table 4).

The creamy white, smooth basidiomata and convex to applanate pileus with striated margin of this *Lactocollybia* resemble those of *L.variicystis*. However, the minute pruinose surface of the pileus, the adnexed to adnate and close to subdistant lamellae, plicate-sulcate striations rather than translucent, much larger hymenial and tramal gloeocystidia, and mostly fusoid gloeocystidia in lamellae demarcate *L.variicystis* from L.cf.epia (Reid and Eicker 1998; Desjardin and Perry 2017; Al-Khesraji et al. 2022; Izhar et al. 2022).

*Lactocollybiagracillima* looks similar to this species with its white basidiomata, translucent striations in margin, and decurrent attachment of lamellae, ellipsoid basidiospores, and presence of gloeohyphal elements in the trama (Pegler 1977). However, pileus with truncated apex, absence of any hymenial gloeocystidia and pleurocystidia, shorter cheilocystidia (23–35 μm), and differently shaped (clavate and often constricted) and shorter (18–35 μm) caulocystidia are the prominent differences (Pegler 1977). The absence of any hymenial gloeocystidia and the smooth margin also demarcate the Chinese *L.subvariicystis* (Hosen et al. 2016).

The water-soaked striated margin of *L.piliicystis* is similar, but the quite longer stipe, smaller basidiospores (5.75–6.2 × 3.2–4.5 μm), and more lanceolate to elongate cylindric with obtuse to mucronate apexed gloeocystidia in lamellae are very different (Reid and Eicker 1998). *Lactocollybiaglobosa* is characterized by its non-hygrophanous, deep umbilicate pileus with an exceptionally inrolled margin and ovoid to subglobose basidiospores (Reid and Eicker 1998).

## ﻿Discussion

*Lactocollybia* is a small genus identified in the field by the typical white and small basidiomata, white lamellae, and dry and pruinose surface. Microscopically, *Lactocollybia* species are characterized by ellipsoid to amygdaliform basidiospores with guttation and the abundance of gloeocystidia in the hymenium and pileipellis. Most species were described based solely on morphology; hence, DNA sequences of holotypes and phylogenetically confirmed samples are very scarce. The scientific names of many public sequences of *Lactocollybia* remain unverified or unidentified. Only two recent studies have proposed nrITS phylogeny with limited public sequences (Hosen et al. 2016; Izhar et al. 2022). In this study, three *Lactocollybia* species are described from Thailand, in which one of them is proposed as a new species, *L.polyhabitata*, while another species matched with the sequences of *L.variicystis* from different countries. Another species, L.cf.epia, has been critically analyzed for its affinity towards *L.epia*. All species in this study belong to the sect. Albae and share a similar appearance, characterized by white to yellowish-white small-sized basidiomata and the presence of gloeohyphal elements. However, the absence of any hymenial gloeocystidia in *L.polyhabitata* easily separates it from L.cf.epia and *L.variicystis*. The translucent striations of the pileus margin, subamygdaliform basidiospores, and ovoid to subcylindrical, inflated hymenial gloeocystidia of L.cf.epia are different from the smooth pileus margin, ellipsoid basidiospores, and cylindrical to subcylindrical hymenial gloeocystidia of the Thai *L.variicystis*.

### ﻿Morphological variations of *L.variicystis*

Morphological characters are often used in species identification and delimitation. However, morphological variation within a single species can be broad and lead to incorrect taxonomic assessments. Recently, *L.variicystis* has been reported from various localities in tropical and subtropical regions. The species was first described from South Africa by Reid and Eicker (1998) and later has been reported in São Tomé (Desjardin and Perry 2017), Pakistan (Izhar et al. 2022), and Iraq (Al-Khesraji et al. 2022). Besides the differences with the type specimen (discussed under notes of *L.variicystis*), we also found variations in some morphological characters among the other specimens. Detailed comparisons have been given in Table 3. Desjardin and Perry (2017) described *L.variicystis* with larger ellipsoid to amygdaliform basidiospores (7.4–9.6 × 5.1–6.4 μm), hymenial gloeocystidia (88–136 × 11–18 μm), cheilocystidia (48–75 × 6.5–14.5 μm), and pileal gloeocystidia (>100 × 9.5–23 μm). The specimen has a yellowish-brown tint at the disc, a pellucid-striate pileus margin, close spacing of lamellae with 2–3 series of lamellulae, and is found in coastal forests. Pakistani and Iraqi specimens have ellipsoid to amygdaliform basidiospores, large hymenial gloeocystidia and cheilocystidia, and the absence of caulocystidia (Al-Khesraji et al. 2022; Izhar et al. 2022). Additionally, the light pink lamellae, plicate-sulcate striate margin, and lamellae spacing in Pakistani *L.variicystis* are quite unlike our described *L.variicystis* (Izhar et al. 2022). Overall, the major distinguishing features between the other *L.variicystis* specimens and Thai specimens include a striated pileus margin, close to subdistant lamellae spacing, lamellulae in 1–3 series, yellow or golden hymenial gloeocystidia, quite larger gloeocystidia in the hymenium and pileipellis, absence of any pigment in the pileipellis, larger cheilocystidia, prominent presence of pleurocystidia (except the type specimen), and caulocystidia (only in African specimens). These morphological variations from different geographical collections raise questions about intraspecific variability and potential environmental effects on phenotypic plasticity.

**Table 3.** Comparative study between all the documented *L.variicystis*.

Names	*L.variicystis*	*L.variicystis*	*L.variicystis*	*L.variicystis*	*L.variicystis*
**Description**	Reid and Eicker (1998)	Desjardin and Perry (2017)	Izhar et al. (2022)	Al-Khesraji et al. (2022)	This study
**Country**	South Africa	São Tomé, Central Africa	Pakistan	Iraq	Thailand
**Habitat**	*Salix* sp. stump	Decaying wood in coastal forest with cacao and banana.	Bark of *Psidiumguajava* and *Vachellianilotica*	*Prunusarmeniaca* dead wood	*Dipterocarpus* sp.
**Molecular data**	No	Yes	Yes	Yes	Yes
**Pileus size**	2–18 mm	8–22 mm	14–29 mm	up to 15 mm	5–15 mm
shape	Convex then flattened	Broadly convex to plano-convex, sometimes with a low, broad umbo	Reniform broadly convex to plano-convex, slightly depressed at disc	Spherical, convex to flattened, with or without central depression	Convex to planoconvex
color	Not mentioned	White overall or with a pale yellowish brown disc	White, pale yellow when bruised	White, creamy, yellowish brown becoming creamy white	White to yellowish white changing to greyish yellow on drying
surface	Smooth	Smooth	Smooth	Smooth	Smooth
margin	Slightly striate or plicate	Pellucid-striate	Plicate-sulcate striate	Striations not mentioned/absent	Striations not present
**Lamellae**	Adnexed	Ascending-adnate	Adnate	Adnate	Adnate
color	Creamy white	White with concolorous edges, developing yellowish brown stains where bruised	Light pink	White to creamy white	White
spacing	Not mentioned	Close to subdistant	Close to subdistant	Close to subdistant	Crowded
lamellulae series	Not mentioned	2–3	1–2	Not mentioned	6
furcation	Sometimes forked	Rarely forked	Not mentioned/absent	Not mentioned/absent	Absent
**Stipe size**	32 × 1–1.8 mm, 3 mm wide at base	5–12 × 1–2 mm	10–15 × 5–8 mm	20–50 × 1–2 mm	7.5–22.5 × 1.5–2.5 mm
attachment	Not mentioned	Central to eccentric	Central to eccentric	Central to eccentric	Central to slightly eccentric
color	Creamy white	White when young, becoming yellowish brown	White to pale yellow	Creamy white	White to yellowish white
surface	Smooth and pruinose	Minutely pruinose	Minutely pruinose	Smooth or very finely pruinose	Smooth
**Basidiospores**	6.6–8 × 4–6 μm	7.4–9.6 × 5.1–6.4 μm	(6.4–)7.4–9.4(–9.8) × (4.8–)5.2–5.8(–6.2) μm	5–8.75 × 5–6.5 μm	(3.1)4.4–*5.4*–6.9 × (2.3)3.1–*3.9*–4.5(5.1) μm
shape	Broadly amygdaliform with one conspicuous oil droplet (uni-guttulate)	Ellipsoid to broadly amygdaliform	Broadly ellipsoid to ellipsoid, some amygdaliform, multi-guttulate	ellipsoid to amygdaliform, with one large oil droplet (uni-guttulate)	Ellipsoid, rarely subglobose, uni-guttulate
**Hymenial gloeocystidia**	40 × 9 μm	88–136 × 11–18 μm	53–105 × 9–23 μm	80–150 × 10–15 μm	24–32.6 × 3.2–6.2 μm
shape	Cylindric to subcylindric	Clavate to fusoid-ventricose, obtuse	Clavate, some ventricose with obtuse apex	Fusiform, claviform, rounded	Cylindrical to subcylindrical with mostly obtuse to sub-capitate apex
color	Yellowish	Yellow to golden	Not mentioned	Yellow or golden	Yellowish brown
**Pleurocystidia**	Mentioned as the gloeocystidia	Versiform, majority cylindrical-capitate	Narrowly cylindrical, versiform, cylindrically capitate	Cylindrical, subcylindrical, lageniform	Absent
**Cheilocystidia**	17–40 × 3.1–4.5 μm	48–75 × 6.5–14.5 μm	47.5–76.5 × 6.5–15.5	30–50 × 6.5–10 μm	22.4–34.3 × 4.4–6.8 μm
shape	Lageniform, cylindric or fusiform to lanceolate, or with a swollen base contracted into long undulating neck; obtusely rounded or capitate apex	Versiform, narrowly cylindrical to fusoid, ventricose or lageniform, sometimes capitate	Narrowly cylindrical to fusoid, ventricose, sometimes lageniform, capitate	Cylindrical, subcylindrical, lageniform	Cylindrical to subcylindrical with mostly obtuse to sometimes sub-fusoid, sub-capitate to mucronate apex
**Caulocystidia**	Similar to cheilocystidia but smaller	Similar to the cheilocystidia, versiform	Not mentioned/absent	Not mentioned	Absent
**Pileipellis**	Cutis, hyphae 4–11.6 μm wide, interspersed with elements of gloeosystem	Cutis, radially arranged hyphae 2.5–5 μm wide, interspersed with elements of gloeosystem	Cutis, radially arranged hyphae 2.5–5 μm wide interspersed with elements of gloeosystem	Not mentioned	Cutis, interspersed pale brownish pigment, hyphae 2.4–4.5 μm wide, interspersed with elements of gloeosystem
gloeocystidia in pileipellis	150 × 11.6 μm	>100 × 9.5–18 (–23) μm, versiform, fusoid-ventricose to clavate	110–132 × 5–14 μm, narrowly clavate, versiform	Not mentioned	26.5–65 × 7.6–10 μm, lageniform to fusoid

**Table 4.** Comparative study between all the documented *L.epia*, *L.angiospermarum*, L.cf.epia, and *L.polyhabitata* from the current study.

Name	*L.angiospermarum*	*L.angiospermarum*	*L.epia*	*L.epia*	*L.epia*	*L.epia*	L.cf.epia	*L.polyhabitata*
**Description**	Original description (Singer 1948)	Pegler (1977)	Original description (Pegler 1986)	Yang (2000)	Cortez and Sulzbacher (2009)	Senthilarasu and Kumaresan (2016)	Current study	This study
**Country**	USA	East Africa	Sri Lanka	China	Brazil	India	Thailand	Thailand
**Pileus size**	10–24 mm	10–55 mm	10–25 mm	10–30 mm	7–42 mm	10–20 mm	4–28 mm	6–16 mm
shape	Convex, umbonate or papillate	Convex to planoconvex, umbonate or slightly depressed	Sometimes obtusely umbonate or depressed to sub umbilicate	Convex to applanate, sometimes slightly centrally depressed	Planoconvex with slightly depressed	Convex	Broadly convex to planoconvex to applanate without any umbo or depression	Planoconvex to applanate
surface	Glabrous but margin initially pubescent	Smooth, glabrous	Smooth, glabrous	Smooth, glabrous	Smooth	Smooth, glabrous	Smooth	Minutely pruinose
margin	Striate	Translucent-striate	Not striate to finely striate when moist	Not found	Striate	Translucent-striate	Translucent-striate up to 2/3^rd^ of the pileus	Not striate
**Stipe**	15–25 × 1–3 mm	20–80 × 2–5 mm	10–40 × 2–4 mm	10–60 × 2–5 mm	19–56 × 1.5–4 mm	10–25 × 1–2 mm	10–15 × 1.5–2.5 mm	10.4–15.7 × 1.3–2.2 mm
**Basidiospores**	7.5–9 × 5.5–6 μm	7.5–11.5 × 3.2–6 μm	7.5–9.5 × 3.5–5 μm	6–9 × 4–6 μm	7.5–11 × 4.2–5 μm	6–8 × 3–4.5 μm	5.3–8.4 × 3.9–4.7 μm	6.3–10.6 × 3.4–4.9 μm
shape	Subfusoid-ellipsoid	Elongate ellipsoid, amygdaliform	Ellipso-piriform to subamygdaliform	Subamygdaliform	Amygdaliform	Broadly amygdaliform	Broadly ellipsoid to subamygdaliform	Ellipsoid to oblong
**Gloeohyphal elements**	Not reported	45–230 × 5–12 μm	45–230 × 5–12 μm	Not reported	55–92 × 6.5–12 μm	Not reported	23.2–67 × 6–11.5 μm	22–95 × 8–19.2 μm
shape		Elongate fusiform	Elongate fusoid hyphal segments		Fusiform		Attenuated at both ends	Attenuated at both ends
presence	Context, hymenophoral trama, cutis, and stipe	Context, hymenophoral trama, cutis, and stipe	Context, hymenophoral trama, cutis, and stipe		Context, hymenophoral trama, cutis, and stipe		Context, hymenophoral trama, cutis, and stipe	Context, cutis, and stipe
content	Intensely greenish (pileipellis)	Greenish yellow	Greenish yellow		Greenish		Yellowish	Yellowish
**Pleurocystidia**	Not reported	Not found	Not found	Not found	Not found	Not reported	45.1–58.5 × 6.9–9.1 μm, cylindrical with obtuse to sub-mucronate apices	Not found
**Cheilocystidia**	Not reported	24–50 × 4–10 μm	24–45 × 3–10 μm	20–38 × 5–9 μm	23.5–43 × 6–8.5 μm	Not reported	23.4–53.5 × 4.1–7.6 μm	15.6–36.6 × 2.5–6.2 μm
shape		Cylindric-clavate with subcapitate apices or ventricose	Cylindrico-clavate with few contents	Subcylindrical to sub-fusiform	Subcylindrical to lageniform with subcapitate to capitate apices		Similar to pleurocystidia with crystalline content	Variable, usually subcylindrical, subclavate to lageniform with obtuse to sub-capitate apices with few content
**Hymenial gloeocystidia**	Stated as numerous	25–37 × 4.5–9 μm	25–35 × 5–8 μm	30–65 × 8–18 μm	24–38 × 5–10 μm	Not reported	17.8–28.9 × 6–12.9 μm	Not found
shape		Clavate fusiform with mucronate apices	Clavato-fusoid with mucronate apices	Fusiform, clavate or cylindrical	Fusiform with mucronate apices		Ovoid to subcylindrical, inflated, or clavate, sometimes subventricose or subfusoid	
content		Greenish yellow	Subhyaline to greenish-yellow	Yellow to yellow brown	Greenish		Yellowish	
**Pileipellis**	Cutis	Unpigmented cutis with hyphae 3–9 μm	Unpigmented cutis with hyphae 3–9 μm	Not reported	Cutis with hyphae (8–29 μm diam.) with fine encrustations	Not reported	Unpigmented cutis with hyphae 4.2–6.9 μm	Unpigmented cutis with hyphae 1.7–3.2 μm wide

Before molecular studies, minor morphological differences often suggested species delimitation; in the type description of *L.variicystis* (Reid and Eicker 1998), it was noted to be very similar to *L.globosa*, mainly differing in its growth habit (scattered versus large troops). Such phenotypic differences debate whether the two specimens are distinct species or interbreeding populations within a species (Petersen and Hughes 1999). The geographical isolation or local adaptation can cause such phenotypic plasticity (West-Eberhard 1989), resulting in the phenotypic variation of the segregated population being broader while the genotype remains unified. The type specimen of *Lactocollybiavariicystis* from South Africa (Reid and Eicker 1998), the São Tomé collection (Desjardin and Perry 2017), and the recent specimen from Pakistan (Izhar et al. 2022) showed similar tropical habits to the Thai specimens, whereas the Iraqi *L.variicystis* has a temperate host association (Al-Khesraji et al. 2022). Despite all specimens sharing the typical morphology, such as white to cream basidiomata, ellipsoid to amygdaliform spores, and the abundance of hymenial cystidia and gloeocystidia, variations, especially in the size of cystidia, can occur in individual collections. In this study, we found notable differences in the Thai specimens, such as a non-striate pileus margin, lamellae spacing with different lengths of lamellulae, a smaller size of gloeocystidia, and the absence of pleurocystidia and caulocystidia (Table 3).

In the nrITS phylogeny (Fig. 1), the lack of resolution of the branches gave it the appearance of a single cohesive group or a complex clade representing *L.variicystis*. However, the short branch lengths can also suggest the occurrence of rapid speciation events that resulted in little genetic divergence among lineages. Our specimens are nested in a large clade with São Tomé, Pakistani, and Iraqi specimens (90% bootstrap support) (Fig. 1). This concludes that these sequences belong to *Lactocollybia* and can be identified as *L.variicystis*. The inconsistent identification may be a result of the superficial similarity and the overlooking of some microscopic characters.

The morphological variations among different geographical samples could be a signal for the differentiation of cryptic species within a species complex. This incident may infer rapid diversification or limited genetic divergence during space and time. To ascertain these assumptions, molecular verification of the type material, multi-gene analyses, morphological study, and ecological data of undescribed sequences and additional samples are required. Considering the current data, our Thai collections are hence identified as *L.variicystis*.

### ﻿The case of *Lactocollybiaepia/L.angiospermarum*

*Lactocollybiaepia* was originally described from Sri Lanka by Pegler (1986). Based on the morphological similarity, another species, *L.angiospermarum* Singer, first reported from Florida, USA (Singer 1948), was synonymized with *L.epia* (Pegler 1986), suggesting a pantropical distribution. The occurrence of *L.epia* in parts of Africa was also inferred from the herbarium specimens of *L.angiospermarum* by Reid (1975), Pegler (1977), and Reid and Eicker (1998). The protologue of *L.angiospermarum* (Singer 1948) and *L.epia* (Pegler 1986) indicates extensive morphological overlap between both species. Consequently, multiple authors have treated *L.angiospermarum* as a synonym of *L.epia* (Pegler 1986; Reid and Eicker 1998; Yang 2000). However, this conclusion has not been validated at the molecular level due to the absence of confirmed sequences for both species. Based on the currently available morphological data, we concur that *L.angiospermarum* may be considered a synonym of *L.epia*. Furthermore, in our phylogenetic analysis, the sequences designated as *L.angiospermarum* did not fall in L.cf.epia clades. The absence of any validated publication of these sequences further contributes to whether these sequences truly represent *L.angiospermarum*/*L.epia*.

#### ﻿Lactocollybiacf.epia

When morphological characters of two of the Thai specimens were compared with all the relevant literature of *L.epia* and *L.angiospermarum* (Table 4), certain variations were noted. The basidiospores of Thai *Lactocollybia* are found to be smaller in size than the other collections: *L.angiospermarum* from East Africa (7.5–11.5 × 3.2–6 μm) (Pegler 1977), *L.epia* from South Africa (6.2–9 × 4.2–5 μm) (Reid and Eicker 1998), and Sri Lanka (7.5–9.5 × 3.5–5 μm) (Pegler 1986). *Lactocollybiaepia* has also been reported from China (Yang 2000), Brazil (Cortez and Sulzbacher 2009), and India (Senthilarasu and Kumaresan 2016). The Chinese specimen morphologically differs by its changing to brownish upon drying, longer and wider stipe (10–60 × 2–5 mm), larger (30–65 × 8–18 μm) and differently shaped (mostly fusiform) hymenial gloeocystidia, absence of pleurocystidia, and shorter (23.4–53.5 × 4.1–7.6 μm) cheilocystidia (Yang 2000). The Brazilian samples have larger basidiomata (pileus 7–42 diam., stipe 19–56 × 1.5–4 μm), differently shaped cheilocystidia, gloeocystidia, and larger basidiospores (7.5–11 × 4.2–5 μm) (Cortez and Sulzbacher 2009). The description of *L.epia* from India has mostly macromorphological characters and basidiospores (Senthilarasu and Kumaresan 2016). Specimens from India may appear similar to our species; however, the absence of key morphological details and molecular data prevents confirmation of this similarity. Interestingly, the morphological characteristics of all previously described *L.epia* and *L.angiospermarum* align closely, showing minimal variation. However, the complete absence of pleurocystidia and shorter gloeohyphal elements distinguish the Thai specimens. In the phylogenetic analysis (Fig. 1), two of our sequences (PQ530290–PQ533676) are clustered with sequences labelled as *L.epia* from various geographical regions. All these sequences are from undescribed specimens with no morphological data. In the nrITS phylogeny (Fig. 1), other sequences submitted as *L.epia* did not exhibit strong phylogenetic affinity to our sequences and are placed in phylogenetically distinct clades. Sequences MW445913 and KU320581 from India as *L.epia* formed a separate clade distantly related to our collections, whereas ‘*L.epia*MN523272’ from China formed a lineage with ‘*L.subvariicystis*ON715776’ from Australia. Since none of the sequences have any substantial taxonomic assessments, the authenticity of these sequences as *L.epia* is difficult to conclude. Due to the lack of confirmation regarding the authentic clade representing *L.epia* and based on the morphological differences observed in the Thai collections, we cannot confidently align these specimens with *L.epia*. Therefore, we have decided to leave them unnamed and designate the clade as L.cf.epia 1. The clade bearing the Indian sequences as *L.epia* has been named as L.cf.epia 2.

#### ﻿*Lactocollybiapolyhabitata*

Phylogenetically, our samples (PQ530288–PQ530289) clustered with two sequences designated as *L.angiospermarum* along with other unidentified sequences (Fig. 1). Although these sequences were collected from the same location as the type (Florida, USA), they have not been validated by any publication or description confirming the authenticity of the name. Regardless of whether *L.angiospermarum* is a synonym of *L.epia* or not, *L.polyhabitata* exhibits noticeable morphological variation when compared to the protologue of both species (Table 4). The absence of gloeocystidia in the hymenium is particularly significant, as most *Lactocollybia* species possess them. In the original description of *L.angiospermarum* (Singer 1948), hymenial gloeocystidia were described as “numerous,” so their absence in *L.polyhabitata* makes this species quite unique. In *Lactocollybia*, species are often distinguished by such clear and simple morphological characters. Additionally, the yellowish content (greenish to greenish yellow in *L.angiospermarum/epia*) of gloeohyphal elements and oblong basidiospores are in contrast. *Lactocollybiapolyhabitata* is clearly distinct from both *L.epia* and *L.angiospermarum* based on these key characters. Thus, this species represents a new and separate species based on the morphological distinction.

### ﻿Intercontinental conspecificity

The geographical distribution of mushroom species remains poorly understood due to insufficient data and sampling. The analyses of DNA sequences of mycorrhizal and saprotrophic fungi often provide evidence of regional geographic distribution rather than intercontinental distribution. The study of Bazzicalupo et al. (2019) suggested that the distribution range of the majority of species could be up to ~ 4,000 km, while only a few species could have a broad distribution, most of which have been associated with anthropogenic activity.

Intercontinental conspecificity refers to the occurrence of the same species across different continents in both the southern and northern hemispheres, highlighting the reasons behind such wide geographical distribution. Various factors contribute to assessing such distribution of any taxa, especially in species-rich tropical countries (Vasco-Palacios et al. 2022). The wide intercontinental distribution of several taxa has been reported in previous studies (Wicklow 1981; Lucking 2003; Feuerer and Hawksworth 2007; Galloway 2008; Werth 2011; Allen and Lendemer 2015; Yang et al. 2016; Vasco-Palacios et al. 2022). Further, molecular approaches, along with ecological data, have developed more discrete distribution patterns for many species (Peay et al. 2010; Summerell et al. 2010; Tedersoo et al. 2014; Song and Cui 2017). This has led to the proper identification of many taxa that previously relied solely on morphology or other taxonomic concepts (Vasco-Palacios et al. 2022).

*Lactocollybia* is a saprobic genus and is found on living tree parts (cortex, fallen fruits, etc.) or rotting log stumps or any plant debris (Singer 1986; Reid and Eicker 1998). The members of this genus predominantly inhabit tropical to subtropical climates, with some exceptions in temperate regions [occurrence of *L.cycadicola* (=*L.epia*) with cycads]. The currently accepted species of this genus have been documented worldwide, but the insufficient molecular data of most species hinder the inference of the actual distribution pattern of species in *Lactocollybia*. Based on our findings in this study, all studied species are clustered in clades with public sequences from different geographical localities, indicating that the intercontinental conspecificity may occur more than one time during space and time. In the case of *L.variicystis*, a species originally described from South Africa, its ubiquitous presence in Pakistan, Iraq, the Philippines, India, the USA, the United Kingdom, and more recently, Thailand, is determined by our phylogenetic analyses. Such distribution from tropical climates to temperate climatic zones is uncommon. The presence of conspecific populations across distant geographic regions suggests that *L.variicystis* has either evolved a remarkable adaptability to various ecological niches or has long-distance spore dispersals. However, the more obvious reasons seem to be its preferences for specific tropical to subtropical climates that have facilitated its widespread distribution.

In the nrITS phylogeny (Fig. 1), the closeness of *L.polyhabitata* to the sequences from the countries of both the southern and northern hemispheres refers to its wide geographical distribution. Two Thai samples of the species are conspecific with records from the USA, Australia, and Mexico, respectively. All these records were reported from tropical to subtropical climates. The three USA samples were found in Florida, where the climate varies from subtropical to tropical. The Mexican sample was found in Guadalajara, which has a humid subtropical climate relatively close to a tropical climate. The Australian specimen was reported from Howard Spring Nature Park, which is located in Darwin, Northern Territory of Australia. The region is close to Southeast Asia and has a tropical climate, featuring a wet and dry season.

Thai specimens of Lactocollybiacf.epia from Thailand showed relatedness to specimens from China, Laos, Italy, Russia, and the USA. The Chinese specimens were from Hainan, an island province of China with a tropical climate. The specimen from Italy is from Sicily, which has a typical Mediterranean subtropical climate with hot, dry summers and mild, wet winters. The other *Lactocollybia* sequences are from the USA, Laos, and Russia. Laos shares a comparable tropical climate. The USA specimen is from Ohio, which experiences a humid continental climate, whereas the sample from Russia is from Novosibirsk, which has a continental climate with extreme winters. The closeness with sequences from Italy, Russia, and the USA suggests the intercontinental conspecificity in this species.

These findings refer to these species’ global distribution. Another reason could be the poor knowledge of the ecology of all the *Lactocollybia* species and the species verification using molecular data. The incorporation of a thorough morphological characterization and phylogenetic analyses of all the specimens is essential to confirm the authenticity of all the sequences. We believe that understanding the pattern of intercontinental conspecificity in *Lactocollybia* may provide insight on the evolution and biogeography of saprobic mushroom-forming fungi.

## Supplementary Material

XML Treatment for Lactocollybia variicystis

XML Treatment for Lactocollybia polyhabitata

XML Treatment for Lactocollybia cf. epia

## ﻿Additional information

### Author ORCIDs

Ishika Bera https://orcid.org/0000-0003-0207-3644

Komsit Wisitrassameewong https://orcid.org/0000-0003-1195-0338

Naritsada Thongklang https://orcid.org/0000-0001-9337-5001

### Data availability

All of the data that support the findings of this study are available in the main text.

## References

[B1] Abarenkov K Nilsson RH Larsson KH Taylor AF May TW Frøslev TG Pawlowska J Lindahl JB Põldmaa K Truong C Vu D Hosoya T Niskanen T Piirmann T Ivanov F Zirk A Peterson M Cheeke TE Ishigami Y Jansson AT Jeppesen TS Kristiansson E Mikryukov V Miller JT Oono R Ossandon FJ Paupério J Saar I Schigel D Suija A Tedersoo L Kõljalg U (2024) The UNITE database for molecular identification and taxonomic communication of fungi and other eukaryotes: Sequences, taxa and classifications reconsidered. Nucleic Acids Research 52(D1): D791–D797. 10.1093/nar/gkad1039 PMC10767974 37953409

[B2] Al-Khesraji TO Mezher MA Shugran AH (2022) First report on the morphological and molecular identification of *Lactocollybiavariicystis* from Salahadin Governorate, North Central Iraq. Ecology. Environmental Conservation 28(2): 626–630. 10.53550/EEC.2022.v28i02.008

[B3] Allen JL Lendemer JC (2015) Fungal conservation in the USA. Endangered Species Research 28(1): 33–42. 10.3354/esr00678

[B4] Altschul SF Madden TL Schäffer AA Zhang J Zhang Z Miller W Lipman DJ (1997) Gapped BLAST and PSI-BLAST: A new generation of protein database search programs. Nucleic Acids Research 25(17): 3389–3402. 10.1093/nar/25.17.3389 9254694 PMC146917

[B5] Bazzicalupo AL Whitton J Berbee ML (2019) Over the hills, but how far away? Estimates of mushroom geographic range extents. Biogeography 46(7): 1547–1557. 10.1111/jbi.13617

[B6] Capella-Gutiérrez S Silla-Martínez JM Gabaldón T (2009) trimAl: A tool for automated alignment trimming in large-scale phylogenetic analyses. Bioinformatics (Oxford, England) 25(15): 1972–1973. 10.1093/bioinformatics/btp348 19505945 PMC2712344

[B7] Clark K Karsch-Mizrachi I Lipman DJ Ostell J Sayers EW (2016) GenBank. Nucleic Acids Research 44: D67–D72. 10.1093/nar/gkz956 PMC4702903 26590407

[B8] Cortez VG Sulzbacher MA (2009) *Lactocollybiaepia* (Basidiomycota): Nova ocorrência para o Rio Grande do Sul. Revista Brasileira de Biociências 7(1): 9–13.

[B9] Darriba D Taboada GL Doallo R Posada D (2012) jModelTest 2: More models, new heuristics and parallel computing. Nature Methods 9: 772. 10.1038/nmeth.2109 PMC4594756 22847109

[B10] Desjardin DE Perry BA (2017) The gymnopoid fungi (Basidiomycota, Agaricales) from the Republic of São Tomé and Príncipe, West Africa. Mycosphere 8(9): 1317–1391. 10.5943/mycosphere/8/9/5

[B11] Edler D Klein J Antonelli A Silvestro D (2021) raxmlGUI 2.0: A graphical interface and toolkit for phylogenetic analyses using RAxML. Methods in Ecology and Evolution 12: 373–377. 10.1111/2041-210X.13512

[B12] Feuerer T Hawksworth DL (2007) Biodiversity of lichens, including a world-wide analysis of checklist data based on Takhtajan’s floristic regions. Biodiversity and Conservation 16(1): 85–98. 10.1007/s10531-006-9142-6

[B13] Galloway DJ (2008) Lichen biogeography. In: Nash III TH (Ed.) Lichen Biology.Cambridge University Press, Cambridge, 315–335. 10.1017/CBO9780511790478.017

[B14] Guindon S Gascuel O (2003) A simple, fast, and accurate algorithm to estimate large phylogenies by maximum likelihood. Systematic Biology 52(5): 696–704. 10.1080/10635150390235520 14530136

[B15] Hall TA (1999) BioEdit: a user-friendly biological sequence alignment editor and analysis program for Windows 95/98/NT. In: Nucleic acids symposium series [41 (41)]. Information Retrieval Ltd., London, 95–98.

[B16] Hausknecht A Krisai-Greilhuber I (2008) *Lactocollybiadendrobii* (*Tricholomataceae*, *Agaricales*), a new species from a flower pot in Austria. Österreichische Zeitschrift für Pilzkunde 17: 53–57.

[B17] Hosen MI Li TH Chen XN Deng WQ (2016) *Lactocollybiasubvariicystis*, a new species of little known genus *Lactocollybia* from subtropical south China. Mycosphere 7(6): 794–800. 10.5943/mycosphere/7/6/10

[B18] Hyde KD Norphanphoun C Chen J Dissanayake AJ Doilom M Hongsanan S Jayawardena RS Jeewon R Perera RH Thongbai B Wanasinghe DN Wisitrassameewong K Tibpromma S Stadler M (2018) Thailand’s amazing diversity: Up to 96% of fungi in northern Thailand may be novel. Fungal Diversity 93: 215–239. 10.1007/s13225-018-0415-7

[B19] Izhar A Usman M Bashir H Ashraf S Niazi AR Latif M Khalid AN (2022) First record of *Lactocollybiavariicystis* from Asia. Mycotaxon 137(2): 335–344. 10.5248/137.335

[B20] Kalichman J Kirk PM Matheny PB (2020) A compendium of generic names of agarics and Agaricales. Taxon 69(3): 425–447. 10.1002/tax.12240

[B21] Katoh K Rozewicki J Yamada KD (2019) MAFFT online service: Multiple sequence alignment, interactive sequence choice and visualization. Briefings in Bioinformatics 20(4): 1160–1166. 10.1093/bib/bbx108 28968734 PMC6781576

[B22] Kearse M Moir R Wilson A Stones-Havas S Cheung M Sturrock S Buxton S Cooper A Markowitz S Duran C Thierer T Ashton B Meintjes P Drummond A (2012) Geneious Basic: An integrated and extendable desktop software platform for the organization and analysis of sequence data. Bioinformatics (Oxford, England) 28(12): 1647–1649. 10.1093/bioinformatics/bts199 22543367 PMC3371832

[B23] Kirk P Cannon P Minter D Stalpers J (2008) Ainsworth and Bisby’s dictionary of the fungi (10^th^ edn). Egham, Utrecht, 771 pp. 10.1079/9780851998268.0000

[B24] Kornerup A Wanscher JH (1978) Methuen handbook of colour (3^rd^ edn). Eyre Methuen Ltd., London.

[B25] Lucking R (2003) Takhtajan’s floristic regions and foliicolous lichen biogeography: A compatibility analysis. Lichenologist (London, England) 35(1): 33–53. 10.1006/lich.2002.0430

[B26] Maddison WP Maddison D (2008) Mesquite: A modular system for evolutionary analysis. Evolution 62: 1103–1118.18298648

[B27] Mycoflora of Hawaii (2019) [iNaturalist project]. iNaturalist NZ. https://inaturalist.nz/projects/mycoflora-of-hawaii-2019

[B28] Osmundson TW Robert VA Schoch CL Baker LJ Smith A Robich G Mizzan L Garbelotto MM (2013) Filling gaps in biodiversity knowledge for macrofungi: Contributions and assessment of an herbarium collection DNA barcode sequencing project. PLoS ONE 8(4): e62419. 10.1371/journal.pone.0062419 PMC3640088 23638077

[B29] Peay KG Bidartondo MI Arnold AE (2010) Not every fungus is everywhere: Scaling to the biogeography of fungal–plant interactions across roots, shoots and ecosystems. The New Phytologist 185(4): 878–882. 10.1111/j.1469-8137.2009.03158.x 20356342

[B30] Pegler DN (1977) A preliminary Agaric flora of East Africa. Kew Bulletin Additional Series 6: 1–615.

[B31] Pegler DN (1986) Agaric flora of Sri Lanka. Kew Bulletin Additional Series 12: 1–519.

[B32] Petersen RH Hughes KW (1999) Species and speciation in mushrooms: Development of a species concept poses difficulties. Bioscience 49(6): 440–452. 10.2307/1313552

[B33] Prance GT (1987) Etnobotânica de algumas tribos amazônicas. Suma etnológica brasileira 1: 119–133.

[B34] Putzke J (2007) *Lactocollybiaaurantiaca* Singer (*Tricholomataceae*, Basidiomycota): First record from Brazil. Sitientibus. Série Ciências Biológicas 7(2): 161–162. 10.13102/scb8117

[B35] Rambaut A Drummond AJ Xie D Baele G Suchard MA (2018) Posterior summarization in Bayesian phylogenetics using Tracer 1.7. Systematic Biology 67(5): 901–904. 10.1093/sysbio/syy032 29718447 PMC6101584

[B36] Reid DA (1975) Type studies of the larger Basidiomycetes described from southern Africa. Bolus Herbarium. University of Cape Town 7: 1–255.

[B37] Reid DA Eicker A (1998) South African fungi 6. The genus *Lactocollybia* (Basidiomycota) in South Africa. Mycotaxon 66: 153–164.

[B38] Senthilarasu G Kumaresan V (2016) Diversity of agaric mycota of Western Ghats of Karnataka, India. Current Research in Environmental & Applied Mycology 6(1): 75–101. 10.5943/cream/6/2/3

[B39] Singer R (1939) Phylogenie und Taxonomie der Agaricales. Schweizerische Zeitschrift für Pilzkunde 17: 71–73.

[B40] Singer R (1948) New Genera of Fungi-IV. Mycologia 40(2): 262–264. 10.1080/00275514.1948.12017704 20283546

[B41] Singer R (1962) Diagnoses fungorum novorum Agaricalium II. Sydowia 15(1–6): 45–83.

[B42] Singer R (1970) New agarics from South America. Nova Hedwigia 20(3–4): 785–792.

[B43] Singer R (1973) Diagnoses fungorum novorum Agaricalium III. Beihefte zur Sydowia 7: 1–106.

[B44] Singer R (1978) Interesting and new species of Basidiomycetes from Ecuador II. Nova Hedwigia 29(1–2): 1–98.

[B45] Singer R (1986) The Agaricales in modern taxonomy (4^th^ edn). Koeltz Scientific Books, Koenigstein.

[B46] Singer R (1989) New taxa and new combinations of Agaricales (Diagnoses fungorum novorum Agaricalium IV). Fieldiana: Botany New Series 21: 1–133. 10.5962/bhl.title.2537

[B47] Singer R Digilio APL (1952) Pródromo de la Flora Agaricina Argentina. Lilloa 25: 5–461.

[B48] Song J Cui BK (2017) Phylogeny, divergence time and historical biogeography of *Laetiporus* (Basidiomycota, Polyporales). BMC Evolutionary Biology 17(1): 1–12. 10.1186/s12862-017-0948-5 28424048 PMC5397748

[B49] Summerell BA Laurence MH Liew EC Leslie JF (2010) Biogeography and phylogeography of Fusarium: A review. Fungal Diversity 44(1): 3–13. 10.1007/s13225-010-0060-2

[B50] Tedersoo L Bahram M Polme S Koljalg U Yorou NS Wijesundera R Vasco-Palacios A Abarenkov K (2014) Global diversity and geography of soil fungi. Science 346: 6213. 10.1126/science.1256688 25430773

[B51] Vandegrift R Newman DS Dentinger BTM Batallas-Molina R Dueñas N Flores J Goyes P Jenkinson TS McAlpine J Navas D Policha T Thomas DC Roy BA (2023) Richer than Gold: The fungal biodiversity of Reserva Los Cedros, a threatened Andean cloud forest. Botanical Studies 64(1): 1–22. 10.1186/s40529-023-00390-z 37410314 PMC10326184

[B52] Vasco-Palacios AM Lücking R Moncada B Palacio M Motato-Vásquez V (2022) A critical assessment of biogeographic distribution patterns of Colombian fungi. Catalogue of Fungi of Colombia, 121–136.

[B53] Werth S (2011) Biogeography and phylogeography of lichen fungi and their photobionts. In: Fontaneto D (Ed.) Biogeography of microscopic organisms: is everything small everywhere.Cambridge University Press, Cambridge, 191–208. 10.1017/CBO9780511974878.011

[B54] West-Eberhard MJ (1989) Phenotypic plasticity and the origins of diversity. Annual Review of Ecology and Systematics: 249–278. 10.1146/annurev.es.20.110189.001341

[B55] White TJ Bruns T Lee SJWT Taylor J (1990) Amplification and direct sequencing of fungal ribosomal RNA genes for phylogenetics. PCR protocols: a guide to methods and applications 18(1): 315–322. 10.1016/B978-0-12-372180-8.50042-1

[B56] Wicklow DT (1981) Biogeography and conidial fungi. In: Biology of conidial fungi, Vol. 1. Academic Press, Cambridge, 417–447. 10.1016/b978-0-12-179501-6.50021-1

[B57] Wijayawardene NN Hyde KD Dai DQ Sánchez-García M Goto BT Saxena RK Erdoğdu M Selçuk F Rajeshkumar KC Aptroot A Błaszkowski J Boonyuen N da Silva GA de Souza FA Dong W Ertz D Haelewaters D Jones EBG Karunarathna SC Kirk PM Kukwa M Kumla J Leontyev DV Lumbsch HT Maharachchikumbura SSN Marguno F Martínez-Rodríguez P Mešić A Monteiro JS Oehl F Pawłowska J Pem D Pfliegler WP Phillips AJL Pošta A He MQ Li JX Raza M Sruthi OP Suetrong S Suwannarach N Tedersoo L Thiyagaraja V Tibpromma S Tkalčec Z Tokarev YS Wanasinghe DN Wijesundara DSA Wimalaseana SDMK Madrid H Zhang GQ Gao Y Sánchez-Castro I Tang LZ Stadler M Yurkov A Thines M (2022) Outline of Fungi and fungus-like taxa–2021. Mycosphere 13(1): 53–453. 10.5943/mycosphere/13/1/2

[B58] Yang ZL (2000) Notes on five common but little known higher Basidiomycetes from tropical Yunnan, China. Mycotaxon 74(74): 45–56.

[B59] Yang H Dai Y Xu M Zhang Q Bian X Tang J Chen X (2016) Metadata-mining of 18S rDNA sequences reveals that “everything is not everywhere” for glomeromycotan fungi. Annals of Microbiology 66: 361–371. 10.1007/s13213-015-1116-z

